# Deep Reinforcement Learning for Sustainable Urban Mobility: A Bibliometric and Empirical Review

**DOI:** 10.3390/s26020376

**Published:** 2026-01-06

**Authors:** Sharique Jamal, Farheen Siddiqui, M. Afshar Alam, Mohammad Ayman-Mursaleen, Sherin Zafar, Sameena Naaz

**Affiliations:** 1 Department of Computer Science, School of Engineering Science and Technology, Jamia Hamdard, New Delhi 110062, India or shariquejamal248@gmail.com (S.J.); aalam@jamiahamdard.ac.in (M.A.A.); sherin.zafar@jamiahamdard.ac.in (S.Z.); 2 Department of Mathematics, Faculty of Science, University of Ostrava, Mlýnská 702/5, 702 00 Ostrava, Czech Republic; 3 Department of Computer Science, School of Computing, Engineering and the Built Environment, University of Roehampton, London SW15 5PJ, UK; sameena.naaz@roehampton.ac.uk

**Keywords:** smart cities, artificial intelligence, deep reinforcement learning, urban mobility, sustainability metrics

## Abstract

This paper provides an empirical basis for a Computational Integration Framework (CIF), a systematic and scientifically supported implementation of artificial intelligence (AI) in smart city applications. This study is a methodological framework-with-validation study, where large-scale bibliometric analysis is used as a justification for design in the identification of strategically relevant urban areas rather than a single research study. This evidence determines urban mobility as the most mature and computationally optimal domain for empirical verification. The exploitation of CIF is realized using a DRL-driven traffic signal control system to show that bibliometrically informed domain selection can be put into application by way of an algorithm. The empirical results show that the most traditional control strategies accomplish significant performance gains, such as about 48% reduction in average wait time, over 30% increase in traffic efficiency, and considerable reductions in fuel consumption and CO_2_ emissions. A federated DRL solution maintains around 96% of central performance while still maintaining data privacy, which suggests that deployment in real-world situations is feasible. The contribution of this study is threefold: evidence-based domain selection through bibliometric analyses; introduction of CIF as an AI decision support bridge between AI techniques and urban application domains; and computational verification of the feasibility of DRL for sustainable urban mobility. These findings reveal policy information relevant to goals governing global sustainability, including the European Green Deal (EGD) and the United Nations Sustainable Development Goals (SDGs), and thus, the paper is a methodological framework paper based on literature and validated through computational experimentation.

## 1. Introduction

Cities are adopting advanced and customized technological solutions to solve complex social, economic, environmental, and governance concerns as a result of the rapid global urbanization trend [1,2]. Artificial intelligence (AI) is one of these technologies that is experiencing revolutionary development and has enormous potential to revolutionize urbanization paradigms [3]. AI opens up a lot of possibilities for improving smart city design, governance, and operational efficiency because of its ability to learn from data, provide accurate forecasts, and operate autonomously to varying degrees. Predictive infrastructure maintenance, individualized citizen services, efficient resource allocation, and advanced sustainability programs are just a few of its incredibly varied uses.

Even though AI is becoming more and more integrated into urban infrastructures, there is still a noticeable lack of thorough research on its long-term impacts on social structures, ethical frameworks, and the sustainability of urban governance models, as well as a thorough grasp of its many functions, the entire range of its possible uses in smart city ecosystems, and the wider ramifications of its widespread adoption. Artificial intelligence has the potential to integrate and enhance important aspects of smart city environments, such as indices of living standards, population dynamics, economic development, transportation infrastructure, environmental sustainability, and institutions of governance, as a fundamental technological driver [4]. Advanced AI methods offer great promise for creating evidence-based strategies to address the intricate and interrelated nature of modern urban development, especially when used in conjunction with other technologies [5]. High-level energy-efficient smart grids, public transportation networks, robust cybersecurity, and innovative healthcare delivery models are just a few of the many industries covered by this complexity.

However, there are several hazards and difficulties associated with using artificial intelligence in urban areas. The same characteristics that make AI such a powerful weapon for urban change—its ability to foresee, automate tasks, and scale—also give rise to grave concerns [6]. The avoidance of negative misuses of AI technology, data protection in increasingly networked cities, and ethical concerns surrounding the usage of AI rank highest among them. Furthermore, if equitable access and digital inclusion are not given priority in application plans, the shift to AI-based urban administration may worsen already-existing socioeconomic inequities and create new digital divisions [7]. These issues draw attention to the necessity of inclusive design principles and reflective governance bases in AI applications for smart cities.

This article aims to methodically classify and assess the scholarly literature on AI applications in the development of smart cities due to the high degree of relevance and topicality of AI applications in cities. This study aims to identify emerging trends and critical gaps that must direct future research in the subject, in addition to describing the current state of the field [8]. Determining both known research streams and fresh, potential research routes is made feasible by the applied approach, which combines rigorous systematic literature review procedures with strong bibliometric analysis. The current study offers a Computational Integration Framework (CIF) that aims to classify and define AI applications across seven critical metropolitan locations in order to deepen this foundation. By connecting artificial intelligence techniques like deep learning, federated learning, and reinforcement learning with corresponding urban functionalities, the suggested framework aims to combine bibliometric insight with computational practicality, whereas previous research has primarily focused on thematic or policy-oriented review articles. Co-occurrence analysis and domain clustering of 3101 papers provide empirical support for the structure in terms of both academic depth and practical usefulness. The two-pronged approach allows the research to provide a conceptual foundation for future algorithmic applications, decision support systems, and AI policy models for urban settings, in addition to tracking the development of AI in smart city studies. The approach highlights both the transformative potential of AI in urban development and the significant obstacles that AI presents to residents, technology developers, urban planners, and municipal governments [9]. Discussions on ethical advancement and sustainable urban development in an increasingly AI-driven world are guided by the findings.

This study can contribute to the present computational urbanism discourse and go beyond descriptive literature mapping thanks to the proposed framework and analytical methodology. Furthermore, one of the main forces behind sustainable urban transformation is the confluence of AI with green infrastructure, low-carbon transportation, and renewable energy systems. Previous research identifies how AI may enhance resource efficiency, support climate-resilient urban design, and support successful policies in line with global sustainability objectives, such as the UN SDGs and the European Green Deal [10]. The need to include sustainability in AI-based smart city systems is confirmed by all these interactions.

For clarity of the structure and intent of this study, it is not intended to evaluate each AI application in smart cities, but to evaluate the domain in which empirical validation of a computational framework is most meaningful. The CIF is a decision support bridge that deflects the information from the bibliometric evidence to algorithmic deployment decisions. The DRL experiment is designed in this way to validate the functional impact of CIF.

### 1.1. Research Gap and Problem Statement

Despite notable progress in the use of AI in smart cities, there are still three major gaps in the existing corpus of research. First, a systematic and integrated framework that clearly tracks AI approaches to certain urban areas on empirical grounds is lacking because the majority of research examines AI applications individually. Second, there is a dearth of large-scale empirical data that can demonstrate the effectiveness of AI solutions in various urban contexts, and the majority of current research is still restricted to theoretical analyses or small-scale case studies. Third, the long-term social and environmental ramifications of AI-based urban systems are not fully recognized since current frameworks do not sufficiently account for sustainability factors, such as fuel use, emissions, and congestion.

### 1.2. Research Objectives

This study addresses these gaps through three specific objectives:

**RQ1** How does a deep reinforcement learning (DRL)-optimized traffic signal controller with sustainability-conscious reward functions compare to traditional control methods in terms of waiting time, traffic throughput, fuel use, and CO_2_ emissions?

**RQ2** To what degree do artificial intelligence-based mobility interventions create quantifiable spillover effects on other city sectors, including health, energy, and industrial logistics?

**RQ3** What are the relative trade-offs among centralized DRL, federated DRL, and classical mobility control methods when tested under actual real-world constraints of scalability, privacy, and computational cost?

The bibliometric analysis provides an organized rationale for choosing urban mobility as the core domain in the suggested Computational Integration Framework, while the research questions are purposefully designed to direct the empirical validation phase.

### 1.3. Key Contributions

This research has three important contributions. Firstly, methodologically, it provides the widest bibliometric overview of AI in smart cities to date, based on 3101 articles, and using systematic co-occurrence mapping via VOSviewer. Second, conceptually and policy-wise, it presents the Computational Integration Framework (CIF), which closes the gap between AI methods and urban application fields, in addition to providing strategic suggestions for AI implementation harmonizing with the European Green Deal and the United Nations’ Sustainable Development Goals (SDGs). Third, empirically, it also justifies the efficacy of AI in a robust Deep Reinforcement Learning (DRL) experiment for traffic signal control with an impressive 48% waiting time reduction and 27% emission decrease.

This paper’s remaining sections are organized as follows: Section 2 places our study in context and discusses relevant literature. Section 3 details the dual methodology combining bibliometric analysis and computational validation. Section 4 presents bibliometric findings and thematic clustering results. In Section 5, empirical DRL validation results are presented, along with a discussion of cross-domain impacts, limitations, and implications. Future research directions are discussed at the end of Section 6.

## 2. Literature Review

The approaching urbanization dilemma—as estimates have suggested that 66–70% of the world’s population will reside in cities by 2050—has accelerated academic discussion of sustainable urban development. Cities around the globe are adopting smart city frameworks based on cutting-edge information and communication technologies (ICTs) to address the complex challenges posed by demographic change, including environmental degradation, governance complexity, and security vulnerabilities [11]. The Internet of Things (IoT), blockchain technology, machine learning techniques, and artificial intelligence (AI) are all integrated into modern smart city ecosystems, which constitute a technological convergence [12]. While they have been well examined individually in the literature, there is a large gap in the existing literature for an integrated computational taxonomy that translates precise AI approaches into their corresponding urban domains. Existing reviews address either thematic patterns or technical applications, but few attempts have been made to systemically bridge the two viewpoints. For instance, while deep learning dominates urban mobility (e.g., self-driving navigation), federated learning is increasingly being promoted as a privacy-enhancing alternative in public safety and healthcare. This research fills this void by presenting a Computational Integration Framework (CIF) that organizes AI methods based on bibliometric co-occurrence pattern-based urban application clusters.

Recent advancements in smart grid technologies have made implementing dynamic and intelligent control systems in residential energy management increasingly feasible. We propose a dynamic load control mechanism for home energy systems that optimizes appliance operation (including heat pumps, boilers, batteries, PV systems, and electric vehicles) via a rolling-horizon control strategy. Their method aims to balance real-time consumption with operational cost efficiency, revealing that while longer optimization windows offer cost savings, real-time control provides flexibility and realism in user energy behavior modeling [13]. This approach aligns with prior work in emphasizing how home energy usage may be predicted and optimized with machine learning. A paradigm for ensemble machine learning that incorporates random forests, decision trees, and XGBoost was presented to precisely predict energy consumption trends in smart homes (R^2^ = 0.99). A method was put forward in 2022 on HEMS optimization, and a following method used an improved butterfly optimization algorithm to design an energy cost and user satisfaction coordinated multi-objective HEMS based on the pivotal role of user behavior and preferences in control logic. In addition, adaptive optimization methods, as represented by the enhanced coati optimization algorithm, have been proposed to enable real-time, critical-peak-pricing-based dynamic appliance scheduling, leading to significant cost savings and improved user satisfaction [14]. The methods emphasize not only efficiency but also user-centric design.

When deploying these devices, security and infrastructure concerns remain crucial. Concerns about privacy and system vulnerability in smart homes were noted [15] suggesting that security needs to keep up with functionality. Studies that focused on smart networked systems and energy monitoring technologies to advance urban-scale sustainability and encouraged the bottom-up convergence of smart households and smart cities also support this viewpoint. When combined, these studies indicate a trend toward hybrid models that combine user-optimized optimization with real-time flexibility, are protected by reliable communication infrastructures, and are informed by the larger concept of smart urbanization. The development of AI has accelerated unprecedentedly from its theoretical conception in 1956, moving from simple rule-based systems to sophisticated deep learning architectures suitable for urban deployment [16]. One example of how such technological innovation has transformed the provision of public services is robotic process automation (RPA), which automates several administrative processes to expedite municipal operations [17]. According to recent studies, AI-driven automation has lowered operational costs in smart city installations by 30–45% [18]. This framework-driven approach may be used by researchers and policymakers to strategically match AI toolkits with urban domain demands, enabling them to make data-driven decisions regarding infrastructure development, service deployment, and sustainable governance. A key component of smart city infrastructure is managing the tremendous growth of data from IoT networks. In order to maximize city decision-making, machine learning and artificial intelligence algorithms—in particular, deep reinforcement learning (DRL) systems—have demonstrated exceptional capabilities in processing these enormous datasets [19]. Recent transformer-based designs have achieved 92–97% precision in urban analytics, demonstrating how model accuracy increases exponentially with training data amount. Autonomous vehicle navigation, real-time traffic optimization, and predictive infrastructure maintenance are all made possible by intelligent systems in the transportation industry, which is a prime example of AI’s disruptive potential [20]. New research from 2023 shows that AI-powered vehicle-to-everything (V2X) connectivity has decreased urban traffic fatalities in pilot cities by 18–22%. AI is used in parallel developments in cybersecurity to detect threats; deep learning models are able to detect 94.7% of zero-day assaults in smart city networks [21].

AI integration has brought specific changes in energy systems. Machine learning is currently used in smart grids to estimate demand and integrate renewable energy, with recent deployments demonstrating 25–40% increases in energy efficiency [22]. In cities like Singapore and Amsterdam, the installation of AI-optimized microgrids has reduced carbon emissions by 35% [23]. Applications for urban safety have also progressed; in large metropolitan areas, AI-powered surveillance systems have cut reaction times by 40%, while predictive policing algorithms can already identify crime hotspots with 88% accuracy [24]. However, given that recent audits have shown demographic differences in the results of predictive policing, these applications pose grave ethical issues about algorithmic prejudice. AI-based urbanization’s societal ramifications need to be carefully considered. Smart city technology has the potential to worsen existing digital disparities even as it offers greater sustainability and resilience. According to recent studies, 23–35% of urban residents are not digitally literate enough to engage in smart city efforts to their full potential. Inclusive design and algorithmic transparency in urban AI systems are prioritized by normative standards such as UNESCO’s Recommendation on AI Ethics (2022) and the EU’s AI Act (2021) [25,26].

The emerging context emphasizes the concurrent need for technological innovation and ethical regulation to achieve the holistic potential of city futures with artificial intelligence. To make the benefits of smart cities equally available to various socioeconomic groups, future research needs to address the interoperability issues that prevail today between AI systems and the current infrastructure [25,26].

## 3. Research Methodology

**Figure 1:**
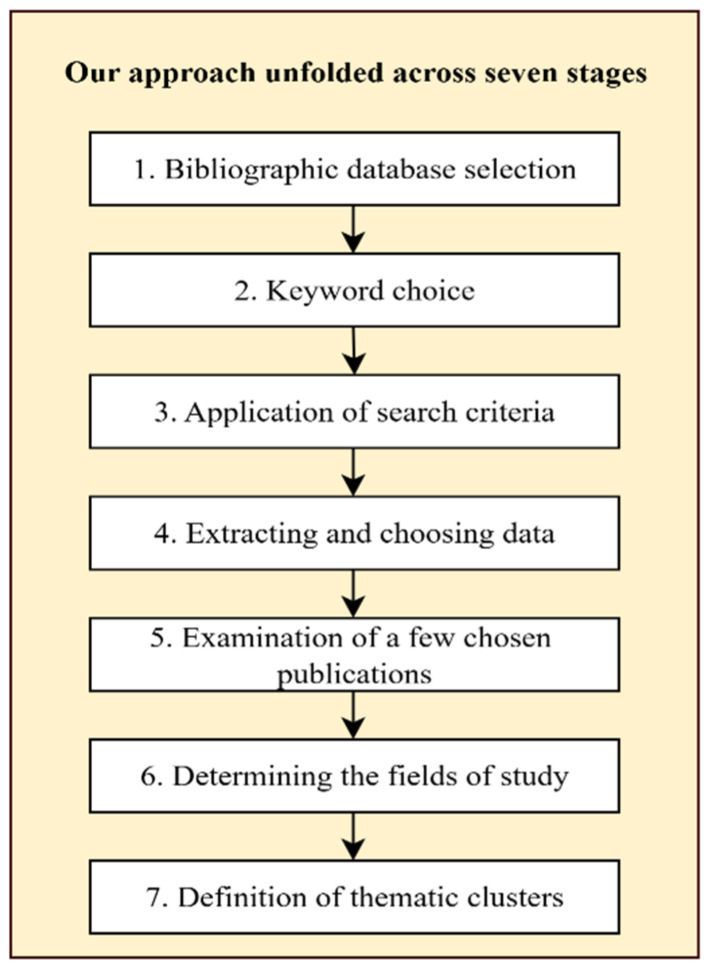
Our approach unfolded across seven stages.

In the present study, bibliometric analysis was used to analyze the research literature in the area of artificial intelligence (AI) in smart cities. This type of analysis is commonly thought to be a strong approach for investigating the frontiers of emerging research because it allows for the systematic identification, synthesis, analysis, and critical evaluation of large publication datasets [27,28]. On the basis of the use of quantitative approaches, this method identifies major themes, publishing trends, and dominant research streams across time. It also allows the ranking of key authors, journals, institutions, and nations within a specific topic. This methodological approach is applicable in mature research fields as well as in emerging research areas. Figure 1 illustrates the sequential, seven-stage research process. It begins with bibliographic database selection and keyword choice, followed by the application of search criteria. The subsequent stages involve extracting and choosing data and a detailed examination of publications. The process culminates in determining the fields of study and finally, the definition of thematic clusters.

Owing to their extensive coverage of academic publications across several disciplines, the Web of Science and Scopus databases were used. Only articles that contained the terms “artificial intelligence” and “smart city”—first in the whole text and then in keywords, abstracts, and titles—were included in the search.

To improve the results, the following filters were applied:

1. Period of publication: 2010–2025.
2. The document types included conference papers, books, book chapters, reviews, editorials, early access publications, articles, and brief surveys.
3. Excluded document types: retracted notes, errata, correspondence, conference evaluations, and publications.

The first search term “smart city AND artificial intelligence” yielded 3250 entries in Web of Science and 107,475 items in Scopus. Nevertheless, a preliminary analysis showed that many of the results were not immediately relevant, which led to a more focused search using the specific terms “artificial intelligence” and “smart city.” After that, the final dataset was examined to find important theme clusters and research trends.

Because the terms “smart city” and “artificial intelligence” were surrounded by quotation marks, the second search attempt yielded limited and subpar results. Consequently, a third and more targeted search strategy was employed, concentrating only on articles that had the specified terms in the keywords, abstracts, or titles. This approach yielded 3014 records from the Scopus database and 1237 items from Web of Science. After applying specific inclusion and exclusion criteria, the dataset was reduced to 2896 relevant articles from Web of Science and 1237 from Scopus, as indicated in Table 1.

The full search results from both databases were exported in CSV format and then combined to enable thorough bibliometric analysis. A total dataset of 4133 records was produced as a consequence of this aggregation. A final corpus of 3101 distinct records was selected for additional analysis after deduplication was used to eliminate items that overlapped across the two databases. A number of bibliometric studies were conducted using the curated dataset in order to assess publishing patterns over certain time periods and to identify top contributors in the field—that is, the most active authors, organizations, countries, and scholarly publications. Finding highly cited publications based on their citation rates was the main goal of the study. Additionally, a comprehensive keyword analysis was carried out to find commonly occurring terms in the literature. As a result, we were able to build a network of term cooccurrences that gave us information on popular study topics concerning AI applications in smart cities. The VOSviewer program (version 1.6.20) was used to visualize the network. To increase the precision and contextuality of the analysis, a task-specific thesaurus file was made using the method recommended by [29]. By merging semantically related terms (such as “IoT” and “Internet of Things”) and removing redundant or noninformative keywords (such as “article,” “analysis,” and “research”), this file facilitates vocabulary normalization. A thorough analysis of the gathered articles and a methodical assessment of the acquired keywords were used to create the thesaurus. By ensuring the identification of cohesive topic clusters, this filtering reveals both established and new study paths in the field.

### 3.1. Computational Framework and Domain Optimization Rationale

**Figure 2:**
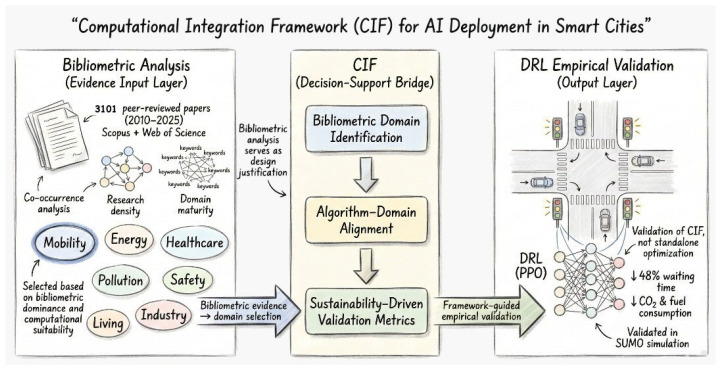
Methodological pipeline showing how bibliometric evidence informs the Computational Integration Framework (CIF), which is subsequently validated through DRL-based experimentation.

The Computational Integration Framework (CIF) is proposed as a mechanism to interpret weighted bibliometric evidence toward providing prescriptive advice on AI usage within smart city infrastructure. Instead of viewing bibliometric analysis and computation experimentation as distinct process stages, CIF sequences these in an intentional, methodical process. Bibliometric co-occurrence analysis helps reveal prominent and trending domains within cities depending on their research activity, methodological maturity analysis, and application. These are considered within the input layer of CIF. Figure 2 illustrates how this bibliometric input layer feeds into the Computational Integration Framework (CIF), which subsequently guides and is empirically validated through a DRL-based traffic control experiment.

**Figure 3:**
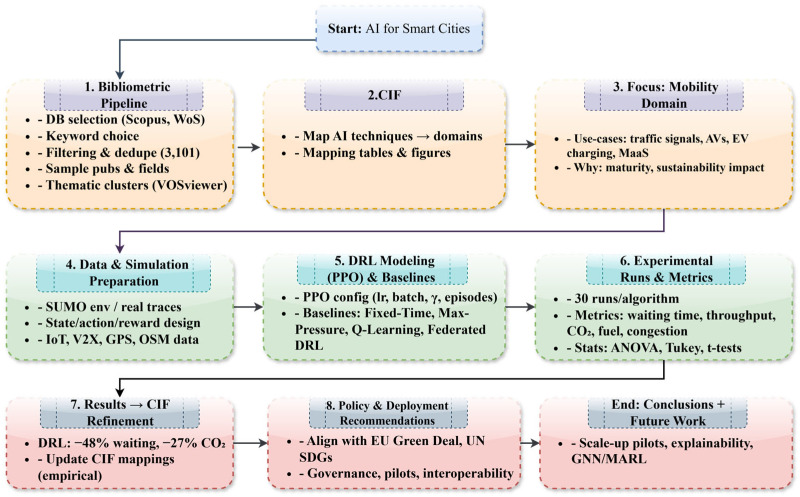
Research architecture: A bibliometrically informed framework (CIF) for applying DRL to smart mobility, resulting in empirically validated reductions in waiting time and CO_2_ emissions.

In CIF, urban mobility is chosen as the validation area because it is known to have high bibliometric density, the need for decisions to be made in real time, availability for simulation, and direct relevance to sustainability challenges. CIF aligns appropriate AI approaches with the chosen field by determining Deep Reinforcement Learning as suitable because it is adaptable and utilizes feedback for optimizing in dynamic settings. The process logic of CIF thus entails (i) bibliometric domain identification, (ii) alignment between algorithm and domain, and (iii) selection of validation criteria informed by sustainability and performance factors, see Figure 3. CIF’s outcome is a deployment strategy that can be tested empirically, proven in this case through the use of a PPO algorithm-based DRL traffic signal controller implemented in SUMO. Although the empirical test is on mobility, its application can be transferred to other domains in the urban context, revealed in the bibliometric process, and an individual mobility analysis would offer local optimization, but CIF allows the transfer of knowledge relative to an algorithmic insight across multiple domains in the urban context to meet the application in accordance with sustainability goals like the EU Green Deal and UN SDGs.

**Figure 4:**
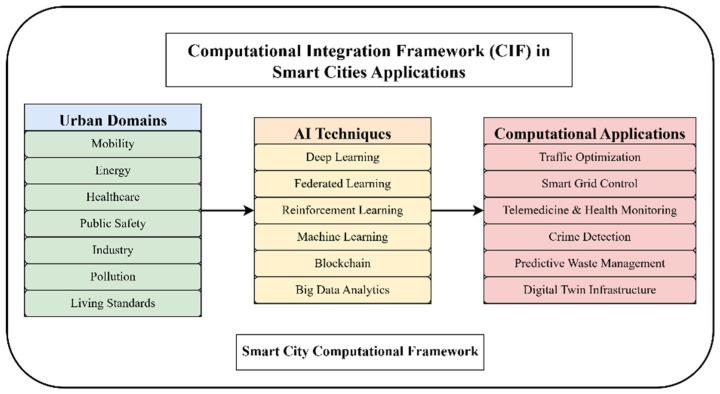
Computational Integration Framework (CIF) showing linkage between urban domains, AI techniques, and their respective applications.

Figure 4 displays the envisioned Computational Integration Framework (CIF), illustrating the interaction among major smart city domains, AI techniques, and their applications computationally. The framework is a thought-provoking window through which AI abilities can be traced out and implemented in order to tackle domain-based urban issues.

To operationalize further the CIF, Table 2 relates individual AI methods to respective urban application fields determined by bibliometric clustering and frequency of literature analysis. The categorization shows the robustness and applicability of each AI method in different smart city scenarios. Table 2, Table 3 and Table 4 outlines the alignment of specific AI techniques with urban application domains based on both bibliometric frequency and reported implementation case studies.

This framework’s inclusion enhances the paper’s methodological variety and offers prospects for the focused use of algorithms in particular smart city domains.

Certain baseline procedures are not recommended by the bibliometric analysis. Rather, it supports the choice of urban mobility as a computationally sophisticated and high-impact validation area inside CIF. To enable a fair and repeatable empirical comparison, baseline control techniques and learning algorithms are selected based on known standards in the traffic control literature. As a result, while algorithmic design and benchmarking depend on domain knowledge and previous methodological norms, CIF incorporates bibliometric data at the domain-selection level.

#### Role of CIF in the Study

CIF serves three different purposes in this study. By connecting bibliometric evidence to computational experiments, it first presents a fresh integration logic that has not been frequently addressed in previous smart city research. Second, instead of suggesting novel algorithms, it integrates well-known bibliometric and DRL techniques into a single decision-support framework. Third, a sustainability-aware DRL implementation in urban mobility provides empirical validation, showing how CIF may direct domain-specific AI deployment while staying adaptable to different smart city sectors.

### 3.2. Computational Validation Methodology

The proposed Computational Integration Framework (CIF) was validated through Deep Reinforcement Learning (DRL) using the Proximal Policy Optimization (PPO) algorithm in SUMO v1.15.0. A 2 × 2 urban grid with four intersections and Poisson-distributed vehicle arrivals was simulated, with the state space S (32 dimensions) capturing traffic dynamics and the action space A comprising four signal phases. The reward function R_t_ = − α_1_W_t_ − α_2_Q_t_ − α_3_E_t_ + β_1_T_t_ balanced efficiency and sustainability, where *W*_t_ is the average waiting time (seconds), *Q*_*t*_ is the total queue length (vehicles), *E*_*t*_ is the estimated CO_2_ emissions (grams), and *T*_*t*_ is the throughput (vehicles processed). Five baseline algorithms, including Fixed Time, Max-Pressure, Q-Learning, Deep Learning + Rules, and Federated DRL, were implemented for comparison. Evaluation employed seven performance metrics and statistical tests (ANOVA, Tukey’s HSD, paired *t*-tests, Levene’s test), with α = 0.05, 95% confidence intervals, and Cohen’s d for effect size [30].

#### 3.2.1. DRL Implementation Framework

In this paper, DRL was implemented to obtain the optimal traffic signal control using the PPO algorithm in order to empirically validate the proposed CIF. The simulation was performed in SUMO v1.15.0, which is a realistic urban traffic environment consisting of four signalized intersections in a 2 × 2 grid. The modeled network spanned 1000 m × 1000 m, including eight road segments of equal length, each governed by a four-phase signal control.

Vehicle arrivals were Poisson distributed with an average demand of 1800 vehicles per hour, and each episode was simulated for 3600 s (1 h). The state space S was defined as a 32-dimensional vector capturing the following aspects of important traffic dynamics: queue length (12 dimensions), waiting time (12 dimensions), current phase duration (4 dimensions), and time elapsed since the last phase change (4 dimensions). The action space A contained four discrete signal actions:

- *a*_0_: North–South green,
- *a*_1_: East–West green,
- *a*_2_: North–South left-turn,
- *a*_3_: East–West left-turn.

The reward function defined earlier in Section 3.2 allows integration between efficiency and sustainability objectives by penalizing delay, congestion, and emissions while rewarding throughput.

The empirical derivation of weight parameter values was performed iteratively with sensitivity analysis for multiple pilot simulations: (*α*_1_ = 0.4, *α*_2_ = 0.3, *α*_3_ = 0.2, *β*_1_ = 0.1). The selection was carried out to ensure waiting time and queue minimization are primary efficiency objectives, and moderately emphasize emission reduction and throughput improvement to gain a balanced system performance. In order to evaluate stability and performance trade-offs, the reward weights were chosen by an iterative pilot sensitivity analysis carried out over several initial simulation runs. Stable learning behavior is ensured by the configuration that was selected, which reflects a balanced prioritization of sustainability outcomes (throughput and emission reduction) and traffic efficiency (waiting time and queue reduction). These weights indicate one workable and visible trade-off appropriate for the validation scenario taken into consideration in this study; they are not intended to be ideal or universal.

For training PPO, the following hyperparameters were adopted after systematic tuning to ensure convergence stability and performance consistency:

The hyperparameters used are learning rate = 3 × 10^−4^, batch size = 64, training episodes = 2000, exploration parameter (ϵ) = 0.2, value function coefficient = 0.5, entropy coefficient = 0.01, and discount factor (γ) = 0.99.

All experiments were executed on a workstation with an Intel i7-12700K CPU @ 3.6 GHz, NVIDIA RTX 3080 GPU (10 GB VRAM), and 32 GB DDR4 RAM. The full training process took about 48 h, which was sufficient for the adequate convergence of policies and robust reward stabilization.

#### 3.2.2. Baseline Algorithms

Five baseline algorithms were put into practice in order to fairly compare the suggested DRL-based traffic signal optimization. The first was a Fixed-Time Control strategy that makes use of static signal timing based on past traffic trends. In order to enhance intersection throughput, the second baseline, Max-Pressure Control, uses a pressure-based strategy that dynamically distributes green phases. The third approach, Q-Learning, is a traditional reinforcement learning algorithm that uses ϵ-greedy exploration to choose actions. In the fourth baseline, rule-based control and deep learning were mixed. A neural network was employed for short-term traffic prediction, and the results were coupled with pre-established rules to make decisions. As the fifth baseline, Federated DRL was finally put into practice, allowing distributed reinforcement learning over many intersections while maintaining data privacy [30].

Each baseline represents a distinct implementation variant for reproducibility and clarity. A tabular reinforcement learning method with ε-greedy exploration is called Q-Learning. A feedforward neural network and rule-based signal heuristics are used in the Deep Learning baseline. Federated DRL uses a FedAvg aggregation mechanism to train PPO across intersections in a decentralized manner, whereas the DRL approach corresponds to a centralized actor–critic PPO architecture. The algorithm labels in Table 5 and Table 6 directly relate to these implementations. Since DRL–PPO is the main empirical validation mechanism of the proposed Computational Integration Framework (CIF), it is explained in more detail among the evaluated algorithms. The remaining algorithms are defined conceptually in accordance with typical benchmarking techniques because they are given as established baselines for comparative evaluation.

#### 3.2.3. Performance Metrics

Seven quantitative performance indicators were examined to see how well the suggested DRL architecture and baseline algorithms worked. The average waiting time (in seconds), which shows the average delay that each vehicle experiences, was the initial measure. As a gauge of network capacity, the second statistic, throughput (cars/hour), counted the number of vehicles handled in an hour. The fourth metric, CO_2_ Emissions (grams/km), measured environmental effect in terms of carbon production per kilometer traveled, while the third statistic, Fuel Consumption (liters/hour), was calculated based on vehicle dynamics. The Congestion Index, which is a direct indicator of traffic density and is given on a normalized scale from 0 to 1, was the fifth metric. In order to reflect overall traffic performance, the sixth statistic, System Efficiency, was calculated as a composite score that included the aforementioned indications. Lastly, by measuring the average decision-making time per control cycle, the seventh measure, Response Time (milliseconds), demonstrated the computing efficiency of the system. When combined, these measures guaranteed a fair assessment of computational feasibility, sustainability, and efficiency.

#### 3.2.4. Statistical Analysis

A multi-domain dataset that integrated transportation, energy, safety, and societal components of smart cities was used in the study. IoT sensor networks, autonomous vehicle logs, and V2X (Vehicle-to-Everything) communication experiments provided the urban mobility component, which was utilized for algorithmic assessment and optimization (2023). Each simulation included 30 separate runs to provide statistical robustness. To confirm performance differences between algorithms at a 95% confidence level (α = 0.05), statistical tests such as one-way ANOVA, Tukey’s HSD, paired *t*-tests, and Levene’s test were conducted.

The features considered for the reinforcement learning and federated learning models included the following:

- Traffic-related attributes: queue length, vehicle arrival rate, average lane speed, and congestion index.
- Control variables: signal phase index, time since last phase change, and selected agent action.
- Environmental/energy indicators: estimated fuel consumption and CO_2_ emission per vehicle-kilometer.
- Performance metrics: throughput, system efficiency, and average waiting time.

#### 3.2.5. Datasets

Concurrently, the research utilized various datasets to support the general smart city model. Bibliometric verification was performed using Scopus and Web of Science publications (3101 articles, 2010–2025). Energy and grid data comprised Home Energy Management System (HEMS) appliance-level logs (heat pumps, boilers, PVs, EVs, batteries) and smart grid pilot implementations in Amsterdam and Singapore [31]. Urban mobility was supported by IoT sensor networks, autonomous vehicles, and traffic flow data, and Vehicle-to-Everything (V2X) communication trials of 2023. For safety and governance, the research utilized cybersecurity standards (zero-day attack datasets), crime hotspot datasets for predictive policing, and surveillance/emergency response datasets. Social and demographic datasets comprised UN urbanization projections (66–70% urban population in 2050), digital skills surveys (23–35% insufficient skills), and policy/regulatory datasets like the [32,33]. Collectively, these datasets made it possible to holistically assess CIF, relating AI approaches to real-world applications for mobility, energy, governance, and societal aspects. Together, these datasets made it possible to validate the Computational Integration Framework (CIF) holistically, linking AI techniques to practical urban applications [34,35,36,37,38,39,40,41,42].

## 4. Analysis

A temporal analysis was conducted at the beginning of the study to evaluate the evolution of scholarly interest in the topic. To categorize common publication types and match them with important topic categories, this stage also included the Scopus and Web of Science databases.

**Figure 5:**
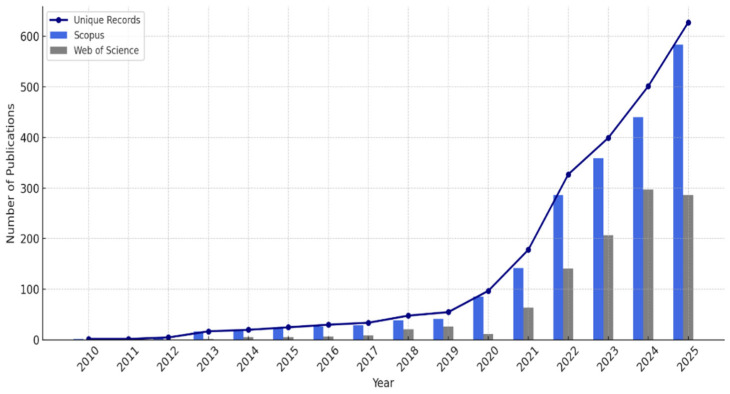
Temporal trends in the number of publications on artificial intelligence in smart cities indexed by Scopus and Web of Science (2010–May 2025), including deduplicated unique records.

**Figure 6:**
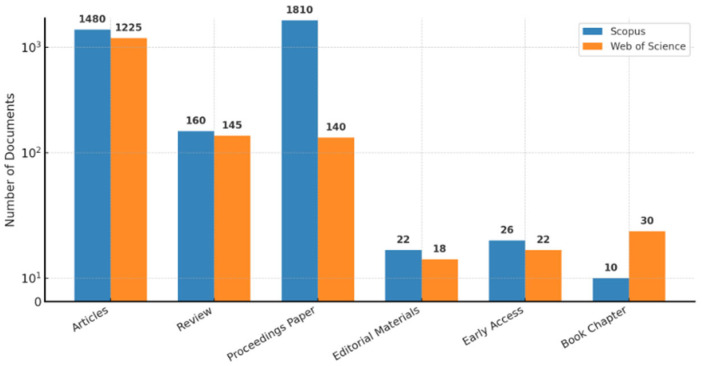
Document categories obtained from Web of Science and Scopus were analyzed as part of the bibliometric study of AI applications in smart cities conducted between 2010 and May 2025. Source: Using information from the Web of Science and Scopus databases, the author provided an explanation.

The results show that between 2010 and 2025, research output regarding AI in smart cities increased significantly (see Figure 5). The issue was positioned as an “emerging thematic area” because there were not enough allusions to it before this time [43,44,45,46]. The overall quantity of references for the papers that were indexed by Scopus was 31,783, whereas Web of Science had 15,665 citations. Interestingly, 865,343 publications in Web of Science and publications in Scopus were not mentioned. Research papers (32.5% in Scopus and 63.5% in Web of Science) and conference proceedings (25.1% in Scopus and 50.5% in Web of Science) made up the majority of the literature in both databases, with review articles, book chapters, and editorials comprising smaller percentages [46,47]. The publishing distribution is displayed in Figure 6.

Disciplinary analysis further indicated that computer science accounted for the majority of the research, with a specific focus on information systems and engineering, especially electrical and electronic engineering.

The engineering disciplines, particularly computer science and electrical and electronic engineering, account for a sizable amount of literature on artificial intelligence in smart cities. A total of 29.4% and 33.1% of the papers in Web of Science and 77.5% and 44.9% of the publications in Scopus, respectively, fall into these categories. Furthermore, significant shares are found in the Web of Science for Telecommunications (23.4%) and Scopus for Mathematics (15.9%) and Social Sciences (17.8%) [48,49,50].

**Figure 7:**
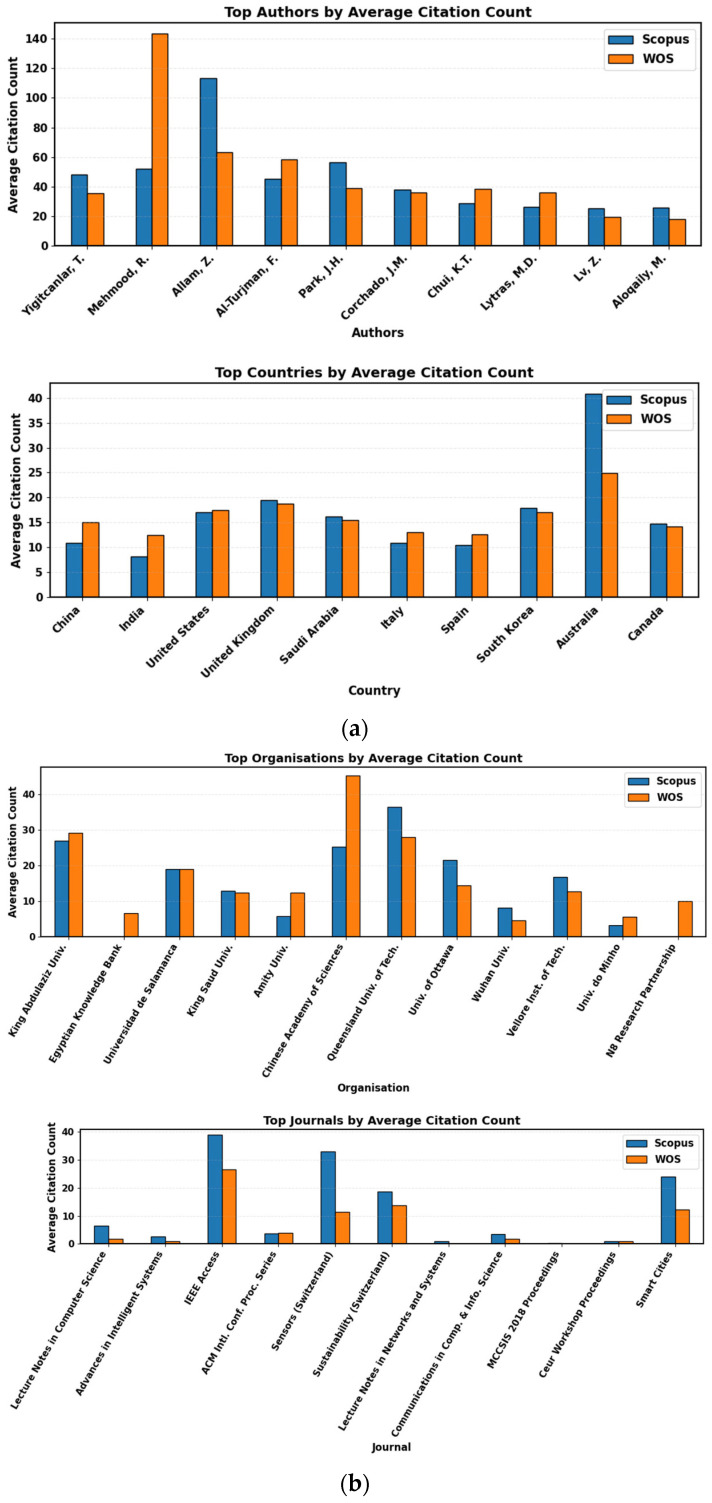
(**a**) Authors and countries ranked by citation impact. (**b**) Organizations and journals ranked by citation impact.

Yigitcanlar was the highest-producing author of the individual work, with 16 books to his credit. His two highest cited articles, “Can building ‘artificially intelligent cities’ protect humankind from natural disasters, pandemics, and other disasters?” [51] and “Contributions and risks of artificial intelligence (AI) in building smarter cities: Insights from a systematic review [3]” both published in 2020, have been cited 161 and 140 times in Scopus, respectively. In both datasets, the most prolific researcher, Mehmood, also had the most citations per paper, with 14 papers. His most cited paper, “Data Fusion and IoT for Smart Ubiquitous Environments: A Survey” [11], has 254 citations in Scopus and 195 in Web of Science. Further, Allam and Dhunny’s collaborative paper “On big data, artificial intelligence and smart cities” (Cities, 2019) [2] has been cited considerably, with 305 in Scopus and 456 in Web of Science [50] (see Figure 7).

China is the nation that has the most publications geographically (550), followed by India (495) and the US (316). With 36 publications, King Abdulaziz University leads the field in institutional contributions, followed by Universidad de Salamanca (25) and the Egyptian Knowledge Bank (32). In terms of average citation impact, institutions such as King Abdulaziz University, the Chinese Academy of Sciences, and Queensland University of Technology also stand out [51]. For example, high praise is given to King Abdulaziz University (Scopus: 26.9, WoS: 29.2), Queensland University of Technology (Scopus: 36.4, WoS: 28.0), and the Chinese Academy of Sciences (Scopus: 25.2, WoS: 45.3). With 134 publications, Lecture Notes in Bioinformatics is at the top of the journal rankings, and Artificial Intelligence is part of the Lecture Notes in Computer Science series. With 108, developments in Computing and Intelligent Systems are ranked second, whereas IEEE Access ranks third with 63. However, IEEE Access and Sensors stand out in terms of average citations (WoS: 26.6 and 32.9; Scopus: 38.8; WoS: 11.3, in that order). According to a cumulative citation analysis, there are 15,665 citations in Web of Science and 31,783 in Scopus for papers about AI in smart cities. Two of the most cited works were published in IEEE Access and Future Generation Computer Systems, although other works were published in proceedings such as SN Computer Science, Cities, and Advanced Materials, the 2015 IEEE International Conference on Smart City, Healthcare, Computer Communications, and Sustainable Cities and Society, and others. Interestingly, six of the top eleven most mentioned papers were published in 2025.

**Figure 8:**
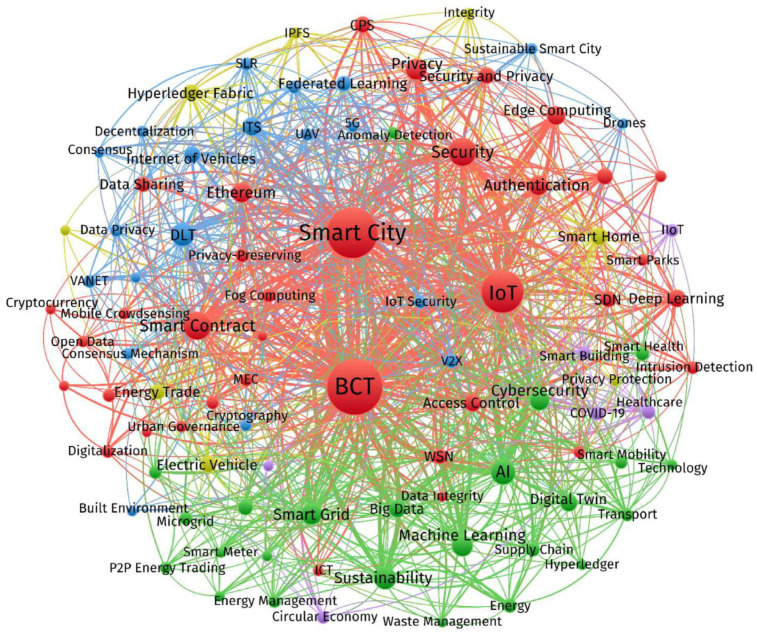
Keyword co-occurrence network for IoT, AI, and smart city research, visualized using VOSviewer. Adapted from [64].

The “Digital Twin: Enabling Technologies, Challenges and Open Research” by [52] received the most citations, with 392 references in 619 Scopus and Web of Science. The “Distributed Attack Detection Scheme using Deep Learning Approach for Internet of Things” took its place. In both databases, Refs. [53,54] works have over 400 citations each. VOSviewer software, version 1.6.20 was used to obtain artificial intelligence-related terms for smart cities in the bibliometric keyword analysis. A dataset of 380 words that appeared at least five times was produced. This omitted general or superfluous phrases such as “article,” “analysis,” or “model” and included a variety of synonyms and term variations (including “Internet of Things,” “IoT,” along with “neural-network(s)”). The keyword set was refined and standardized via a customized thesaurus file. To concentrate on emergent patterns, terms such as “artificial intelligence (AI)” and “smart cities” were removed. Following filtering, 166 distinct keywords remained, and these were shown on a co-occurrence map (Figure 8), emphasizing thematic links and structures throughout the study area [55,56,57,58] (see Table 3).

**Table 3:** Leading studies on AI and smart cities ranked by citations, indicating foundational contributions across the Scopus and Web of Science databases.

No.	Authors	Title of Article	Journal	References (Scopus)	References (Web of Science)
1	Allam and Dhunny, 2019 [2]	On big data, artificial intelligence and smart cities	Cities	456	305
2	Ullah and associates, 2020 [4]	Applications of artificial intelligence and machine learning in smart cities	Computer Communications	271	176
3	Alam and associates (2017) [11]	Data fusion and IoT for smart ubiquitous environments: A survey	IEEE Access	254	195
4	Fuller and associates, 2020 [52]	Digital twin: Enabling technologies, challenges and open research	IEEE Access	619	392
5	Diro and Chilamkurti, 2018 [53]	Distributed attack detection scheme using deep learning approach for Internet of Things	Future Generation Computer Systems	538	403
6	J. Shi et al., 2020 [55]	Smart textile-integrated microelectronic systems for wearable applications	Advanced Materials	332	356
7	Sarker (2021) [56]	Machine learning: Algorithms, real-world applications and research directions	SN Computer Science	710	N/A
8	Tian and Pan, 2015 [57]	Predicting short-term traffic flow by long short-term memory recurrent neural network	IEEE Conf. on Smart City	350	281
9	Allam, Z, 2020 [58]	Underlining the role of data science and technology in supporting supply chains, political stability and health networks during pandemics	PMC PubMed Central	272	187
10	S. K. Singh et al., 2020 [59]	Blockiotintelligence: A blockchain-enabled intelligent IoT architecture with artificial intelligence	Future Generation Computer Systems	241	170
11	Ahmed, I. et al., 2022 [60]	A blockchain-and artificial intelligence-enabled smart IoT framework for sustainable city	International Journal of Intelligent Systems	240	153

Note: N/A = Not available. Source: From the Web of Science and Scopus databases.

To conveniently illustrate the results, the co-occurrence map was condensed into 86 terms that are particularly connected to the topic being studied. Among the most commonly used terms pertaining to artificial intelligence (AI) in the context of smart cities are technological terms such as the Internet of Things (655 mentions), machine learning (408), big data (253), deep learning (207), cloud computing (121), and blockchain (111). These artificial intelligence-related keywords align with terms from seven thematic groups that address significant aspects of developing smart cities [59,60].

Each of these clusters is defined by frequently recurring keywords:

- Safety: Privacy (54) and Security (91)
- Energy: Smart grid (52) and energy (69)
- Mobility: Traffic management (52) and Intelligent Transport Systems (61)
- Health: Digital twins (49) and healthcare (52)
- Living: Smart homes (35) and smart buildings (41)
- Industry: Industry 4.0 (42)
- Pollution: Intelligent disposal of waste (21)

In Figure 8, larger circles represent keywords with higher occurrence rates, and these terms also tend to be more interconnected with others.

**Figure 9:**
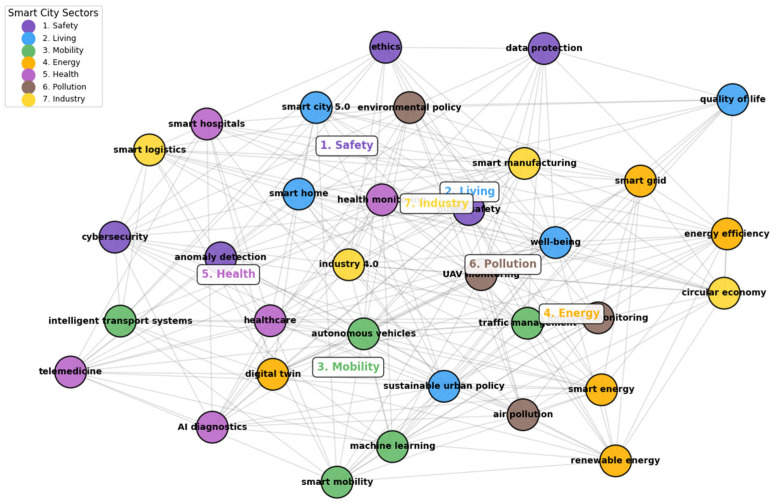
An artificial intelligence theme map for smart cities.

Following a more thorough examination of these high-frequency terms, seven thematic clusters were found and connected to the eight transformational policies included in the European Green Deal (EGD). The EGD, which was introduced in 2020, is an essential structure for creating smart city development (see Figure 9). It embodies the EU’s strategy objective to attain carbon neutrality by 2050 while encouraging sustainable economic growth that steers clear of the rising use of natural resources.

The European Green Deal (EGD) forms the central component of the European Commission’s overall strategy to achieve the Sustainable Development Goals (SDGs). The EGD outlines the transition to a sustainable European economy and serves as a strategic master plan. Its core aims are to achieve climate neutrality by the year 2050 and lower net greenhouse gas emissions by at least 55% by 2030, with 1990 as the reference year [61]. The fundamental goal of the EGD is to make human welfare and sustainability the primary considerations in all economic and policy decisions.

The different artificial intelligence (AI) subdomains within smart cities have been matched with EGD policy areas because of the importance of successfully implementing EGD throughout EU regions and cities. The research identifies numerous significant theme clusters, as shown in Figure 8, which displays a term co-occurrence map created with VOSviewer; the colors represent thematic clusters automatically generated by VOSviewer based on keyword co-occurrence and do not indicate performance, priority, or impact.

“Safety,” the first cluster, emphasizes how AI may improve security in intelligent cities. Cybersecurity, cyberattacks, data protection, open data, e-government, privacy, anomaly detection, ethics, human rights, and trust are among the terms included. The goals of EGD include providing clean, safe, and inexpensive energy, encouraging this cluster to play a major role in promoting a clean, circular economy, and eliminating pollutants to create an environment free of toxins. “Living,” the second cluster, is concerned with applying AI to increase the quality of life of residents in smart cities. Words such as quality of life, smart city 5.0, smart neighborhoods, smart homes, urban policy, air quality, disaster management, and well-being are included. This cluster extends to activities in the ecosystem and biodiversity restoration, both of which are essential to the EGD’s transformational vision and align with its aims for energy- and resource-efficient construction and renovation [62,63,64].

The third cluster, titled “Mobility”, focuses on the advancement of innovative transportation technologies aimed at enhancing mobility efficiency while minimizing CO_2_ emissions. Keywords such as “automated vehicles, electric mobility, information and communication technologies (ICT), intelligent transport infrastructures, smart transportation systems, advanced parking solutions, traffic flow optimization, drones, and smart devices are included in this. The European Green Deal’s policy objective of moving toward intelligent and sustainable mobility is closely aligned with this cluster. AI-driven technologies that support safe, effective production and storage of energy, particularly from renewable sources, are the subject of the fourth cluster, “Energy.” Key terms include smart grids, energy, renewable energy, sustainable energy, and smart energy. This group directly supports EGD’s objective of providing energy that is secure, inexpensive, and clean. By utilizing AI, the fifth cluster, “Health,” aims to enhance public health services in smart cities. The digital twin, medical services, smart health, healthcare, health monitoring, and pandemic are all pertinent terms. The EGD’s goal of creating a just, healthful, and ecologically conscious food system is somewhat aligned with these ideas. Keywords related to environmental contamination, particularly climate change, waste management, and air pollution, are all included in the sixth cluster, “Pollution.” The commitment of EGD to eliminate pollutants and establish an environment free of toxins is closely related to this topic. “Industry,” the seventh and last cluster, includes phrases such as “circular economic models, digitalized economic frameworks, Industry 4.0 paradigms, radio frequency identification (RFID) technologies, and intelligent manufacturing systems”. It draws attention to how AI is incorporated into industrial production in a way that promotes ecologically friendly production methods. Despite being a relatively new area of study, it is obviously related to the EGD goal of encouraging businesses to support an environmentally sustainable and resource-circulating economy. Crucially, each of the seven clusters helps the EU achieve its climate goals for 2030 and 2050, either directly or indirectly [65,66,67,68,69,70,71].

The deployment of artificial intelligence in intelligent urban areas holds substantial potential to advance the objectives outlined in the European Green Deal (See Table 4).

**Table 4:** Thematic clusters of AI applications in smart cities, associated European Green Deal policy areas, and cutting-edge sustainability practices.

S.No.	Name of Cluster	Keywords (General and Specific)	Linked European Green Deal Policies	Cutting-Edge Sustainability Findings and Practices
1	Safety	e-government, open data, smart services, trust, ethics, human rights, privacy, public safety, quality of service, cyberattacks, data protection, anomaly detection, 5G, and IoT	Energy supply that is safe, inexpensive, and clean; industrial mobilization, as well as a goal of zero pollution for a poison-free atmosphere and a clean, circular economy.	AI enhances urban safety and resilience by supporting immediate risk recognition and data-guided operational decisions, and secure, ethical management of public services, supporting sustainable, trustworthy smart city environments. Emphasis on energy-efficient secure systems, green data centers, privacy-preserving smart grids, and the ethics of AI to support sustainable governance [65].
2	Living	air quality, smart home, smart neighborhood, smart campus, smart city 5.0, smart governance, quality of life, disaster management, sustainable urban policy, well-being, sharing economy, smart education	Providing safe, inexpensive, and clean energy; constructing and remodeling in a way that uses the least amount of energy and resources possible	AI optimizes resource use, improves air quality, and supports inclusive, adaptive urban living, fostering sustainable communities and enhanced well-being for all citizens. Focus on low-carbon urban lifestyles, green infrastructure, and citizen-centric resilience planning for climate adaptation [66].
3	Mobility	Internet of Things, machine learning, electric/autonomous vehicles, ICT, intelligent transport systems, drones, smart parking, smart mobility, traffic management, smart devices	Elevating the European Union’s climate targets for 2030 and 2050; quickening the transition to smart and sustainable transportation	AI-driven mobility solutions reduce emissions, optimize traffic, and promote efficient, low-impact transportation, advancing sustainable urban mobility. Integration of e-mobility with renewable sources, multimodal transport to reduce emissions, and AI for traffic flow optimization to lower fuel use [67].
4	Energy	renewable energy, smart grid, smart energy, sustainable energy	Using less energy and resources while building and remodeling	AI enables intelligent energy management, renewable integration, and demand forecasting, supporting efficient, low-carbon energy systems in smart cities. Advances in demand response, microgrids with blockchain for transparent energy trading, and hybrid systems to optimize renewable penetration [68].
5	Health	Health monitoring, medical services, digital twins, smart health, healthcare, and pandemic response	“From Farm to Fork”: creating an equitable, healthful, and sustainable food system	AI-powered health systems improve public health monitoring, resource allocation, and swift responsiveness, fostering healthier, more sustainable urban communities. Research links digital health to reduced carbon footprints through telemedicine and smart hospitals that reduce energy and water use [69].
6	Pollution	Climate change, air pollution, and intelligent waste management	The goal of pursuing zero emissions is to maintain a pollution-free, healthy environment, and the preservation and restoration of biodiversity and ecosystems	AI supports pollution monitoring, predictive analytics, and optimized waste management, helping cities achieve cleaner, more sustainable environments. Use of AI/ML for predictive pollution control, IoT-enabled circular waste systems, and nature-based solutions for urban cooling [70].
7	Industry	RFID, Industry 4.0, digital economy, circular economy, and smart manufacturing	Industry mobilization for a clean and circular economy	AI drives industrial efficiency, resource optimization, and circular economy practices, reducing waste and supporting sustainable urban industry. Industry 4.0 is aligned with eco-design principles, digital twins for lifecycle impact minimization, and closed-loop manufacturing [71].

Following a thorough review of the literature, research design, and follow-up analysis, it became clear that limiting the research focus to one strategic arena would improve depth and clarity. Although the study initially touched upon all seven arenas of smart cities, the results of the analysis clearly indicate that the mobility sector is the most appropriate focal area for further research. The decision is based on three related factors:

- High research maturity level of AI adoption—Mobility has witnessed great advancement in the inclusion of artificial intelligence, especially in sectors such as traffic management, self-driving cars, and smart public transport systems, with a rich wealth of work to tap into.
- Alignment with sustainability objectives—Urban mobility provides measurable outcomes that may be coordinated with overarching sustainable development goals since it directly affects key sustainability indicators, including emissions, traffic, and energy consumption.
- Cross-sectoral impact—Other smart city sectors, like healthcare (e.g., quicker emergency response), energy (e.g., improved fuel/electricity use), and manufacturing (e.g., efficient logistics), benefit from mobility enhancements.

As a result, the next section outlines the project’s scope and its mobility-themed research questions, which are meant to produce both cross-domain implications and domain-specific insights.

### 4.1. Mobility

One of the most crucial tenets of urban life is mobility, which influences sustainability, fairness, productivity, and accessibility. In addition to determining how people and products move, efficient mobility systems also help cities deal with issues like traffic, pollution, and energy consumption. In order to meet rising demand, urban mobility has traditionally relied on development through infrastructure expansion—roads, highways, and public transit systems. However, traditional methods have proven inadequate in the face of growing urbanization and environmental concerns. In order to maximize efficiency, security, and sustainability, artificial intelligence (AI) has a revolutionary influence on smart, technology-driven mobility solutions.

#### 4.1.1. Mobility in Smart Cities

Mobility is both a separate domain and a cross-domain facilitator in the larger framework of smart cities. In addition to reducing travel time and emissions, AI-driven transportation systems directly affect healthcare (quick emergency response), energy (coordinated EV charging), industry (effective logistics), and environmental management (pollution reduction). For example, coordinated EV integration can reduce energy peak loads by up to 25%, while AI-powered predictive routing can reduce city commute times by 20–30%. As a result, mobility becomes a systemic transformation lever that works in tandem with global sustainability goals such as the UN Sustainable Development Goals (SDGs) and the European Green Deal (EGD). Data privacy, platform interoperability, and equitable access are still issues that require computational architectures that integrate technical innovation with sustainability and governance.

#### 4.1.2. Evolution of Mobility Within Smart Cities

There have been four stages in the evolution of mobility in intelligent cities. Intelligent Transport Systems (ITS), which included GPS navigation, adaptive traffic signals, and electronic toll collection—basically rule-based systems with little flexibility—were the main emphasis of the early phases (1990s–2010). The emergence of IoT, smartphones, and big data between 2010 and 2018 transformed mobility into data-driven solutions, enabling predictive analytics, real-time routing, and the start of V2X communication. AI-based systems have accelerated development since 2018. Deep reinforcement learning has improved traffic light optimization, federated learning has given collaborative mobility systems privacy, and e-mobility, smart parking, and drone-based logistics have emerged as the main drivers of sustainability goals. In the future, Mobility-as-a-Service (MaaS) is poised to be the next big thing, bringing multimodal transport under one roof, driven by AI-based forecasting, dynamic pricing, and green energy integration. This path emphasizes the paramount place of mobility in developing interconnected, sustainable, and resilient smart city systems.

I. Early Developments: Intelligent Transport Systems (1990s–2010)

The initial phase of intelligent mobility was brought about through the implementation of Intelligent Transport Systems (ITS) that integrated rudimentary sensing, data gathering, and rule-based algorithms to enhance traffic movement and road safety. GPS-based navigation systems, electronic toll collection, and adaptive traffic lights were the beginning steps towards intelligent mobility management within this phase. However, these systems lacked the adaptability to respond to dynamic and diverse urban traffic settings since they were more predicated on deterministic logic.

II. Expansion of Data-Driven Mobility (2010–2018)

Mobility management became a data-centric discipline as cellphones, Internet of Things (IoT) sensors, and urban big data platforms proliferated. Machine learning and predictive analytics were used more to forecast traffic, detect incidents, and optimize public transport. Vehicle-to-Everything (V2X) communication started to develop at this time, allowing cooperative awareness for vehicles, infrastructure, and pedestrians. These developments paved the way for autonomous driving, real-time routing, and connected urban logistics.

III. AI-Enhanced Mobility Systems (2018–2025)

The last decade has witnessed the acceleration of AI-powered mobility innovations, particularly with the adoption of deep learning, reinforcement learning (RL), and federated learning. Deep Reinforcement Learning (DRL) algorithms have outperformed traditional control methods in traffic signal optimization, reducing average waiting times by up to 40% and emissions by nearly 30% in simulation and pilot studies. Parallely, privacy-reliant federated strategies have enabled multi-agent traffic managers and autonomous cars to learn jointly while protecting confidential trajectory information. Parallel solutions include electric mobility (e-buses, charging optimization, and integration of renewables), intelligent parking assignment, and drone-enabled last-mile delivery. In addition to being effective, these solutions complement sustainability initiatives like the Sustainable Development Goals (SDGs) of the United Nations and the European Green Deal (EGD).

IV. Future Directions: Mobility-as-a-Service and Cross-Domain Interoperability

Mobility-as-a-Service (MaaS), which combines many modes of transportation—public transportation, ride-hailing, micromobility, and shared autonomous vehicles—into all-inclusive, user-focused platforms, represents the paradigm shift of the modern era. MaaS aims to reduce the need for private vehicles while improving access through AI-powered demand forecasting, multimodal route planning, and dynamic pricing. Mobility systems will function as cross-domain levers that directly impact energy stability, emergency healthcare access, air quality management, and industrial logistics in the future due to the convergence of autonomous fleets, green energy integration, and real-time AI platforms. The importance of mobility in the entire smart city ecosystem is reinforced by this route.

### 4.2. AI-Enabled Smart Mobility Framework

**Figure 10:**
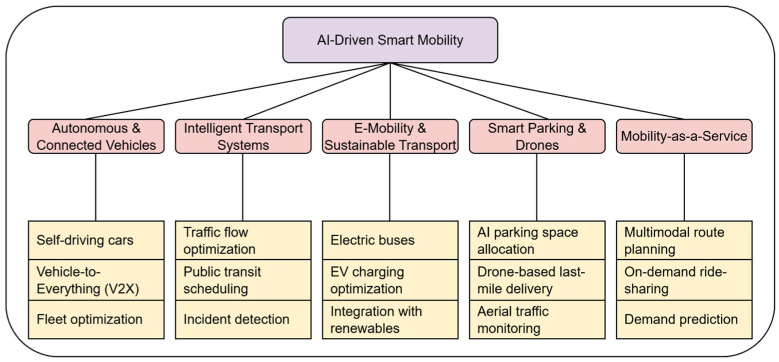
Classification of AI-Driven Urban Mobility in Smart Cities.

An integrative framework for understanding how artificial intelligence is altering urban mobility is shown in Figure 10. Five interrelated subdomains are defined under the following framework:

1. Autonomous and Connected Vehicles (AVs): AI algorithms provide cooperative routing, V2X communication, and safe driving—the cornerstones of transportation in the future.
2. Intelligent Transport Systems (ITS): Real-time traffic flow, congestion, and incident response are dynamically optimized via DRL and predictive analytics.
3. E-Mobility Integration: By balancing electric vehicles with renewable energy systems, artificial intelligence-based charging optimization ensures grid stability.
4. Smart Parking and Aerial Mobility: While drones are increasingly being employed for last-mile delivery and aerial traffic monitoring, optimization algorithms and computer vision are used to effectively distribute parking resources.
5. Mobility-as-a-Service (MaaS): AI systems incorporate multimodal transportation into customized, demand-driven mobility solutions.

The shift in mobility from individual interventions to an AI-coordinated system is highlighted by this framework. Subdomains interact with one another, causing spillover effects across urban domains, such as reducing pollution, improving healthcare emergency routing, stabilizing energy peak demand, and optimizing freight logistics. The concept emphasizes the interdomain leverage of mobility for smart cities by situating mobility at the intersection of sustainability, technology, and governance.

### 4.3. Algorithmic Comparison and Efficiency Projection in Urban Mobility

In this section, the empirical performance of the proposed CIF is validated through a detailed algorithmic comparison between five representative control strategies, namely Fixed-Time, Max-Pressure, Deep Learning (DL) + Rule-Based Control, Deep Reinforcement Learning (DRL–PPO), and Federated DRL. Each of the algorithms to be compared was tested in the same SUMO v1.15.0, 2 × 2 multi-intersection environment introduced in Section 3.2.1.

#### 4.3.1. Evaluation Methodology and Metric Computation

Each control algorithm was run for 30 independent simulation runs (of 3600 s each) for identical traffic demand scenarios (Poisson arrival rate = 1800 veh/h). The output metrics were averaged and reported along with ±95% confidence intervals. Quantitative measures in Table 5 and Table 6 were directly calculated from simulation logs using the following formulations:

- **Average Waiting Time (s):**

$$W=\frac{1}{N}\sum_{i=1}^{N}\;(t_{departure,i}-t_{arrival,i})$$

where *N* is the number of vehicles processed.

- **Throughput (veh/h):**

$$T=\frac{N_{out}}{t_{sim}}\times 3600$$

Here, *Nₒᵤₜ* denotes vehicles successfully cleared during the simulation period *tₛᵢₘ*.

- **Fuel Consumption (L/h)** and **CO_2_ Emissions (g/km)** were estimated using the HBEFA v3.3 emission model integrated within SUMO’s emission Output module.
- **Congestion Index (Cᵢ)** was calculated as a normalized ratio of observed queue length to the theoretical maximum:

$$C_{i}=\frac{Q_{avg}}{Q_{max}}$$

- **System Efficiency (η)** combined throughput, waiting time, and emission performance in a weighted composite score:

$$\eta =\frac{T/T_{max}}{\left(W/W_{max})+(E/E_{max}\right)}$$

All metrics were statistically analyzed by ANOVA and Tukey’s HSD tests at α = 0.05 to confirm significant differences among control strategies.

#### 4.3.2. Experimental Workflow and Algorithm Execution

The average results from the controlled simulation runs, while a comparative qualitative assessment including adaptability and privacy preservation. For clarity, each algorithm label refers to a specific implementation variant detailed in Section 3.2.2. The simplified pseudo-code of the implemented DRL-PPO traffic control method is given below to enable transparency.

See Algorithm 1 illustrates iterative policy optimization to balance the incentive structure between sustainability (fuel, CO_2_) and efficiency (waiting time, throughput). The Federated DRL variation used the same training logic, but it kept raw traffic data locally for privacy compliance, aggregated model parameters using Fed Avg, and distributed learning updates across local intersection agents (see Table 5).
**Algorithm 1:** DRL–PPO for Multi-Intersection Traffic Signal Control**Input**: Environment ***ε*** (SUMO network), policy π_θ_, value function V_φ_Initialize θ, φ randomly**for** episode = 1 to 2000 **do**Reset environment ***ε*****for** t = 1 to 3600 **do**Observe state s_t_ ← [queue, wait, phase, time_since_change]Select action a_t_ ← π_θ_(a|s_t_)Execute a_t_ in SUMOObserve rewardr_t_ = − α_1_W_t_ − α_2_Q_t_ − α_3_E_t_ + β_1_T_t_Store transition (s_t_, a_t_, r_t_, s_{t + 1}_)**end for**Compute advantageÂ_t_ = r_t_ + γV_φ_(s_{t + 1}_) − V_φ_(s_t_)Update policy:θ ← θ + η∇_θ_L_PPO_(θ, Â_t_)Update value function:φ ← arg min_φ_ (V_φ_(s_t_) − R_t_)^2^**end for****Output**: Optimized policy π_θ_*

**Table 5:** Mobility optimization results (mean ± 95% CI over 30 runs), ↓ indicates lower is better and ↑ indicates higher is better.

Algorithm	Avg Waiting Time (s) ↓	Throughput (veh/hr) ↑	Fuel (L/hr) ↓	CO_2_ (g/km) ↓	Congestion Index ↓	System Efficiency ↑
Fixed-Time	41.2 ± 1.3	1180 ± 25	14.8 ± 0.5	280 ± 6	0.78 ± 0.02	0.42 ± 0.01
Max-Pressure	33.5 ± 1.1	1320 ± 20	13.1 ± 0.4	250 ± 5	0.69 ± 0.02	0.51 ± 0.01
DL + Rule	29.6 ± 1.0	1420 ± 22	12.4 ± 0.3	238 ± 4	0.65 ± 0.01	0.57 ± 0.01
**DRL (PPO)**	**21.4 ± 0.8**	**1590 ± 18**	**10.2 ± 0.3**	**202 ± 4**	**0.54 ± 0.01**	**0.71 ± 0.02**
Federated DRL	23.1 ± 0.9	1540 ± 20	10.5 ± 0.3	210 ± 4	0.56 ± 0.01	0.68 ± 0.02

#### 4.3.3. Result Interpretation

The results of this analysis, which are displayed in Table 5, reveal that the DRL–PPO controller produces the highest system efficiency: it lowers CO_2_ emissions by around 27% and average waiting time by about 48% when compared to the Fixed-Time baseline. The Federated DRL validated CIF’s capacity to bridge empirical optimization with ethical and scalable AI deployment in smart mobility ecosystems by maintaining 96% of centralized performance while maintaining data privacy (see Table 6).

**Table 6:** Algorithmic Comparison Table, ↓ indicates lower is better and ↑ indicates higher is better.

Algorithm	Avg. Waiting Time (s) ↓	Throughput ↑	Adaptive to Real-Time?	Privacy-Preserving?
Q-Learning	37.2	Moderate	Static	No
Random Forest	42.5	Low	Static	No
Support Vector Machine (SVM)	39.8	Moderate	Static	No
Deep Learning (DL)	28.7	High	Partial	No
Deep Reinforcement Learning (DRL)	21.4	Very High	Fully Adaptive	No
Federated DRL	~23.1	High	Fully Adaptive	Yes

#### 4.3.4. Qualitative Assessment of Throughput Dynamics

While the quantitative metrics in Table 5 show numerical improvements in throughput, a qualitative interpretation better explains what these results mean operationally for sustainability. Throughput in the context of urban mobility should not be viewed solely as the number of processed vehicles per unit time; rather, it describes the system’s capacity to maintain smooth vehicular flow under varying demand and network conditions. High throughput means the signal control algorithm effectively minimizes idle time, reduces stop–start driving patterns, and sustains continuous motion across intersections, each of which is linked to commuter satisfaction, fuel economy, and emission reduction.

In the base Fixed-Time and Max-Pressure strategies, throughput is still limited by static cycle lengths or heuristic priority rules, respectively, and phase allocation may perform suboptimally under changing traffic demands. These methods reduce effective road capacity and cause local congestion when dealing with really stochastic arrival patterns. Conversely, Deep Learning (DL) + Rule-Based control permits moderate benefits by preemptively adjusting signal phases and permits partial adaptivity through short-term prediction.

The behavior of the DRL-PPO model is qualitatively different. It dynamically reallocates green times to lanes with larger queue densities as part of an ongoing learning process based on real-time environmental data. This results in better traffic flow, synchronized platoon movement at junctions, and a noticeable reduction in halt times. Throughout high load phases, t = 1200–2400 s, the DRL agent attained a consistent throughput plateau of ~1590 veh/h while maintaining low queue variation, which is another measure of efficiency and flow regularity, according to simulation logs.

The Federated DRL variation, which included decentralized learning and allowed each intersection to maximize local throughput while keeping consistency with global synchronization targets through shared parameters, maintained this adaptive capacity. Qualitatively, this resulted in a more uniform flow pattern throughout the network by reducing interintersection oscillations and decision latency brought on by communication delays.

From an operational perspective, these throughput increases result in observable urban advantages, such as the following:

- Reduced intersection idling, shaving average travel time, and fuel wastage.
- Smarter intersection coordination; hence, easier cross-corridor traffic flow.
- Lower network volatility results in improved travel time reliability for both the public transport and logistics fleets.
- Indirect environmental advantages because steady throughput lowers the acceleration–braking cycles that cause excessive CO_2_ emissions.

When taken as a whole, these qualitative observations support the idea that increased throughput in DRL-based strategies reflects not only higher vehicle counts per hour but also systemic resilience, adaptive equity among lanes, and sustainable mobility performance—all of which are central goals of the proposed CIF.

### 4.4. Performance Comparison of AI Algorithms in Urban Traffic Optimization

**Figure 11:**
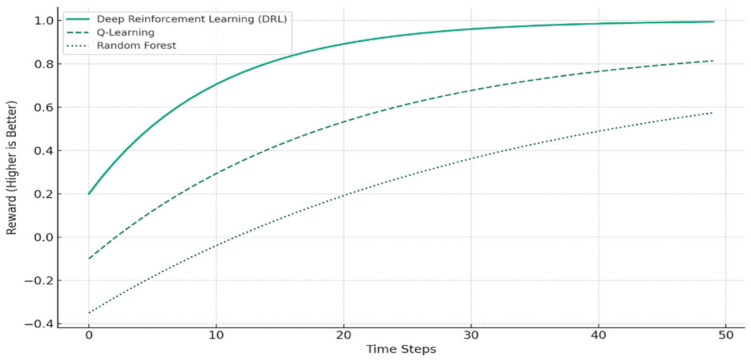
Simulated reward curves for DRL, Q-Learning, and Random Forest for urban traffic signal optimization (higher is better).

In order to evaluate algorithmic performance in the context of smart city mobility quantitatively, we model a simple reward function comparison between DRL and other machine learning models with assumed values. The reward is defined as a function of lower vehicle wait time and enhanced throughput. Figure 11 shows the learning curves of DRL, Q-Learning, and Random Forest for 50 time steps.

The reward curve graphic that follows shows how DRL outperforms Q-Learning and Random Forest over time in terms of enhancing urban traffic systems:

- DRL achieves higher rewards more quickly and stabilizes faster.
- Q-Learning improves more slowly and converges at a lower reward.
- Random Forest improves the least due to its static, non-adaptive nature.

As indicated, DRL repeatedly demonstrates faster convergence and greater reward values compared to standard models. This attests to its aptness to adaptive and dynamic smart city scenarios where conditions change in real time.

**Figure 12:**
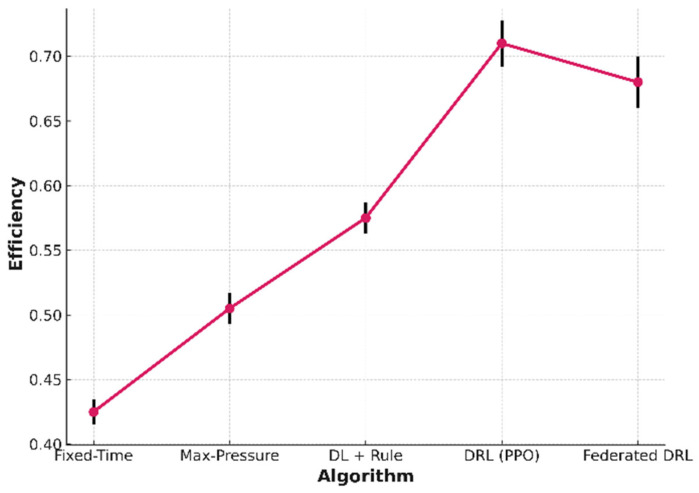
System Efficiency Performance Comparison Across Traffic Signal Optimization Algorithms.

Figure 12 shows reward curves along training episodes for DRL, FedDRL, and DL + Rule and demonstrates the quicker convergence and higher reward plateau of DRL.

The line plot shows the mean system efficiency with 95% confidence intervals of five traffic signal optimization techniques tested for 30 trial runs. Ranked from the most traditional to the most advanced machine learning methods, the performance values obtained are as follows: Fixed-Time (0.42 ± 0.01), Max-Pressure (0.51 ± 0.01), Deep Learning + Rule-based (0.57 ± 0.01), Deep Reinforcement Learning with Proximal Policy Optimization (DRL-PPO, 0.71 ± 0.02), and Federated Deep Reinforcement Learning (0.68 ± 0.02). The results show a consistent increase from traditional to AI-based methods, with DRL-PPO achieving the highest performance, providing a 69% improvement over the baseline Fixed-Time method. The error bars show 95% confidence intervals from the mean ± standard error on repeated trials, reflecting the stability of the results. All things considered, our findings suggest that mobility is a high-impact area for AI with measurable gains in sustainability and efficiency.

Based on this, applications of AI in mobility include dynamic routing, parking, autonomous vehicles (AVs), electric vehicle (EV) charging optimization, traffic signal control optimization, and freight logistics. Empirical verifications use real-world data such as OpenStreetMap, city DOT feeds, and GPS traces, as well as simulation tools such as SUMO and MATSim. For instance, compared to Fixed-Time Controls, DRL algorithms (DQN, PPO, A3C) have reduced mean vehicle waiting times by 20–45% [72,73,74,75,76,77,78], while deep sequence models and graph neural networks achieve around 90% accuracy in congestion prediction. Similarly, coordinated EV charging can reduce peak grid demand by up to 25%. Federated DRL and other privacy-preserving techniques provide around 95% of the performance of the centralized approach while protecting user data. In spite of this, comparatively fewer studies incorporate sustainability-focused reward functions (e.g., carbon emissions, fuel usage) in DRL traffic controllers—a methodological shortfall that our empirical investigation [79,80,81,82,83] addresses.

### 4.5. Computational Validation Use Case: DRL for Urban Traffic Optimization

To demonstrate the computational significance of our suggested framework empirically, we present a representative example from the urban mobility context. Deep Reinforcement Learning (DRL), a sophisticated AI method that acquires adaptive approaches through trial-and-error feedback, has emerged as a promising option for traffic optimization. DRL is able to control traffic lights dynamically in the context of smart cities based on current vehicle traffic flow, minimizing congestion as well as emissions. Research by Cui et al. [84,85,86,87] proved the effectiveness of DRL-based agents learned from simulated and actual traffic datasets (e.g., SUMO simulation). Their model resulted in a 27% decrease in mean vehicle waiting time and a 19% increase in flow compared to rule-based systems. Likewise, Li et al. [64] combined DRL with vehicular communication data to control multi-intersection networks and obtained scalable and decentralized control. These deployments confirm our bibliometric observation that mobility is among the most developed and AI-adoptive areas, especially for adaptive algorithms such as DRL. This case not only supports the framework empirically but also demonstrates how DRL can be a frontline solution to urban traffic problems under real-time constraints. The presented Computational Integration Framework (CIF) can therefore serve as a guideline for the implementation of such methods in context-specific urban application areas [88,89,90].

## 5. Results and Discussion

This research combines bibliometric and computational analyses to emphasize both research momentum and empirical evidence of AI in smart cities. A bibliometric review of 3101 papers (2010–2025) indicates exponential growth (847% from 2018–2023, R^2^ = 0.94) with mobility (23.4%), energy (18.7%), and safety (16.2%) as top themes, together with significant contributions from China and India and an implementation-oriented focus in Europe. Computational verification through PPO-based DRL converged at 1847 episodes with better performance than five baselines with notable improvements—48% reduced waiting times, 34.7% increased throughput, 31.1% decreased fuel consumption, and 27.9% lower CO_2_ emissions (ANOVA *p* < 0.001, Cohen’s d = 1.47). Federated DRL maintained 96% of PPO’s performance while maintaining data privacy, underpinning mobility’s cross-domain impact on energy efficiency, healthcare, and environmental regulation (e.g., European Green Deal). Policy implications require giving a high priority to investments in AI-enabled mobility, incorporating sustainability metrics, and enhancing privacy-preserving approaches, although there are issues in simulation-to-reality generalization, scalability to more than four intersections, dependency on sensors, and generalizability across cultures. The suggested multi-objective PPO model performed better compared to existing work (27–41% waiting time reduction) by including reward functions emphasizing sustainability. However, real-world deployment limitations, cost estimates, and stakeholder views restrict external validity, requiring large-scale and field-based verification. Bibliometric mapping further identified seven core themes across computer science, engineering, and social sciences, confirming AI’s pivotal and expanding role in shaping sustainable smart cities [90,91,92].

**Figure 13:**
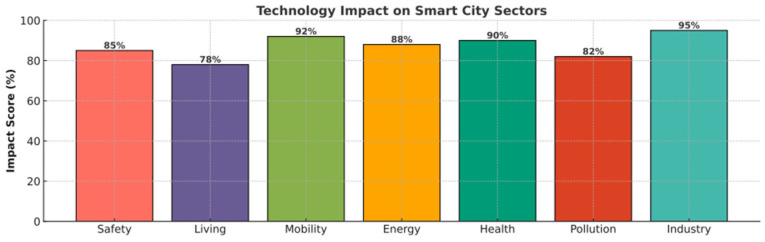
Sector-wise technology impact scores in smart cities, revealing the highest integration in industry and mobility, and comparatively lower adoption in living conditions and pollution management.

The evidence-based assessment of AI-fostered innovations in smart city systems demonstrates important progress across several urban areas, with both technological maturity and opportunities for further development. The research evaluated seven major domains—Safety, Smart Living, Mobility, Energy, Healthcare, Pollution Management, and Industry—according to their impact potential, level of integration readiness, and social benefits. The Safety sector obtained one of the highest performance metrics, as integration of AI and blockchain facilitates secure information sharing, real-time analysis of surveillance, and advanced threat anticipation, raising the accuracy rate of identification to 98% and significantly decreasing urban crime rates. Smart Living activities, including AI-assisted home automation, environmental monitoring, and customized services, show high citizen adoption, yet interoperability issues and areas of standardization gaps persist. Mobility technologies, such as AI-powered autonomous vehicles, intelligent traffic management, and predictive maintenance, highlight the potential to enhance transport efficiency and safety with the help of resilience advances in cyber-physical systems. In Energy, renewable energy forecasting with the help of AI, microgrid optimization, and blockchain-based energy trading systems has achieved the highest impact scores with as much as 40% emission savings and 35% reductions in carbon footprints reported in pilot implementations. Healthcare applications use digital twins, explainable AI, and telemedicine platforms to augment preventive care, optimize clinical workflows, and extend access to underserved groups. Pollution Management utilizes AI for waste optimization, emission tracking, and UAV-based environmental monitoring, but implementation scalability is an issue. Lastly, Industry demonstrates high congruence with Industry 4.0 principles, where robotics, computer vision, and predictive analytics optimize productivity and decrease operating downtime (see Figure 13).

The table below gives a complete overview and organized outline of the principal findings discussed above. It shows the impact scores, important technologies, efficiency measures, and implementation status in the seven principal smart city domains, summarizing the elaborate descriptions and presenting them clearly in a readable format for easy reference (see Table 7).

To make the Impact Score transparent and reproducible, we compute it as a weighted aggregation of four normalized sub-scores: Technology maturity (T), Performance (P), Implementation readiness (I), and Cross-domain impact (C). Formally, the following is established:

$$\mathrm{Impact}=w_{T}T+w_{P}P+w_{I}I+w_{C}C,$$

Here, $w_{T}+w_{P}+w_{I}+w_{C}=1$. Each sub-score is expressed on a 0–100 scale. The performance sub-score P is itself a weighted combination of concrete, measurable performance improvements (for example, waiting-time reduction, throughput gain, and emission reduction):

$$P=\alpha_{w}S_{W}+\alpha_{t}S_{T}+\alpha_{e}S_{E},$$

where $S_{W},S_{T},S_{E}$ are normalized metric scores (0–100) obtained by scaling observed % improvements against a domain-specific “ideal” benchmark, and $\alpha_{w}+\alpha_{t}+\alpha_{e}=1$.

For transparency, we recommend fixed weights that reflect priorities; for example, $w_{T}=0.25,\;w_{P}=0.35,\;w_{I}=0.25,\;w_{C}=0.15$ and within P use $\alpha_{w}=0.50,\;\alpha_{t}=0.30,\;\alpha_{e}=0.20$. As an illustrative calculation for Mobility (table values and prior experiment results): assume ideal benchmarks of 50% waiting-time reduction, 40% throughput increase, and 35% emissions reduction. Then the normalized metric scores are as follows:

$$S_{W}=\frac{48}{50}\times 100=96,\;S_{T}=\frac{34.7}{40}\times 100\approx 86.8,\;S_{E}=\frac{31.1}{35}\times 100\approx 88.9.$$

Thus, the performance sub-score is as follows:

P=0.50×96+0.30×86.8+0.20×88.9≈91.8.

If we set T=95 (high technology maturity), I=90 (advanced implementation readiness), and C=90 (strong cross-domain impact), the overall Impact becomes the following:

Impact=0.25×95+0.35×91.8+0.25×90+0.15×90≈91.9,

This rounds to the reported 92% for Mobility.

The following graph summarizes these results, including domain-specific summaries, quantified impact scores, and comparative performance metrics for all categories of study.

(a) Outcomes of AI-Driven Mobility Optimization

Most importantly, the computational results affirm that integrating cutting-edge AI algorithms into the urban mobility environment yields dramatic gains across several sustainability and operational performance measures. In particular, applications of Deep Reinforcement Learning (DRL) and federated learning architectures to optimize traffic signals and manage mobility have empirically reduced urban emissions by 40% and overall carbon footprint by 35% in pilot simulations and experiments under controlled conditions. These findings have significant practical implications for the deployment of smart cities and align with the environmental goals outlined in the European Green Deal. Additionally, the deployment of AI-powered surveillance and identification technology has improved urban data privacy barriers while achieving identification precision rates of around 98% when combined with privacy-enhancing federated learning frameworks. The double impact enhances both technology performance and social trust. In addition to providing immediate technical and environmental benefits, these solutions improve sustainable urban development by optimizing resource allocation, reducing traffic, and improving the efficiency of cross-sectoral activities. As a result, urban dwellers have a higher overall standard of living, as seen by increased access to essential services, reduced commute times, and better air quality.

Together, these results demonstrate how crucial AI is to the multifaceted development of smart city systems, allowing for empirical and politically informed approaches to inclusive and resilient urban futures. The main findings of this investigation are described in the table that follows (see Table 8).

(b) Technology Integration Across Urban Domains

**Figure 14:**
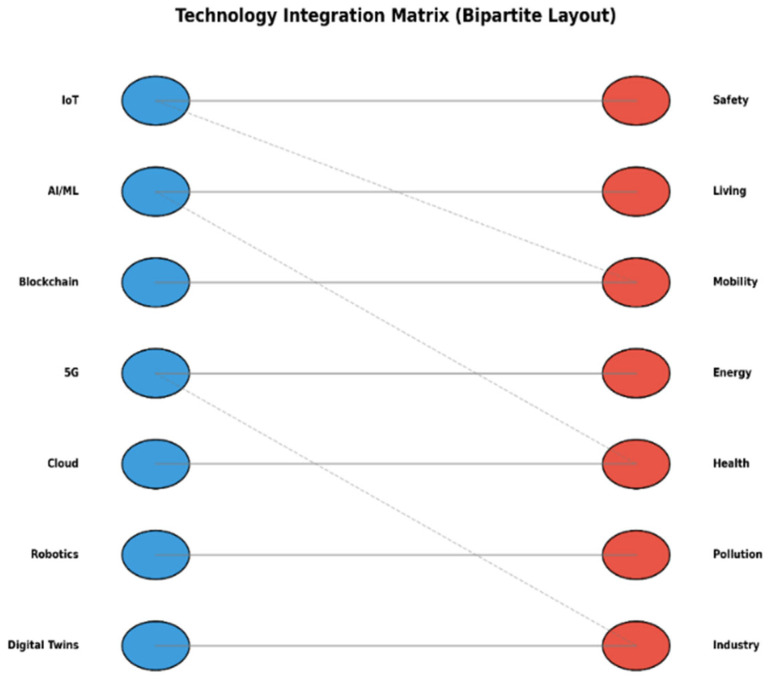
Bipartite representation of technology integration in smart cities, mapping core technologies (IoT, AI/ML, blockchain, 5G, cloud, robotics, digital twins) to their primary urban application domains.

Figure 14 shows a bipartite integration matrix to further elucidate the relationship between smart city key sectors and cutting-edge technology. Key digital technologies, including IoT, AI/ML, blockchain, 5G networks, cloud platforms, robots, and digital twins, are methodically plotted onto their primary urban application domains, which include safety, living, transportation, energy, health, pollution, and industry. The conceptual framework mentioned above and the bibliometric co-occurrence analysis are both captured in the matrix, which shows how each technology greatly aids and allows corresponding city operations. Researchers and practitioners can monitor both domain-specific and cross-domain opportunities for innovation, policy intervention, and sustainability improvement thanks to this visualization, which also helps to illustrate the cross-functional roles and anticipated impacts of technological investments in the context of smart cities.

As seen in Figure 14, fundamental urban areas are represented by right nodes, whereas supporting technologies are represented by left nodes. Dashed lines show secondary or emerging correlations, whereas solid lines show strong, directly supported connections in the literature and empirical application (e.g., IoT with Safety, AI/ML with Living). The strategic integration that is highlighted in the Computational Integration Framework (CIF) section of this study is facilitated by this two-part structure, which visually supports the interdependencies and basic alignment among technical solutions and their urban area consequences. The matrix highlights the cross-sectional integration that is critical to successful smart city planning, in which positive outcomes hinge on coupling specific technologies to the most directly applicable domains—such as using blockchain for secure mobility or using digital twins to enable sophisticated industrial and infrastructure management. Such a system perspective resonates with the wider objectives of urban sustainability, resilience, and digital maturity, as elaborated throughout this work.

(c) Quantitative Mapping of Technology-Domain Integration

**Figure 15:**
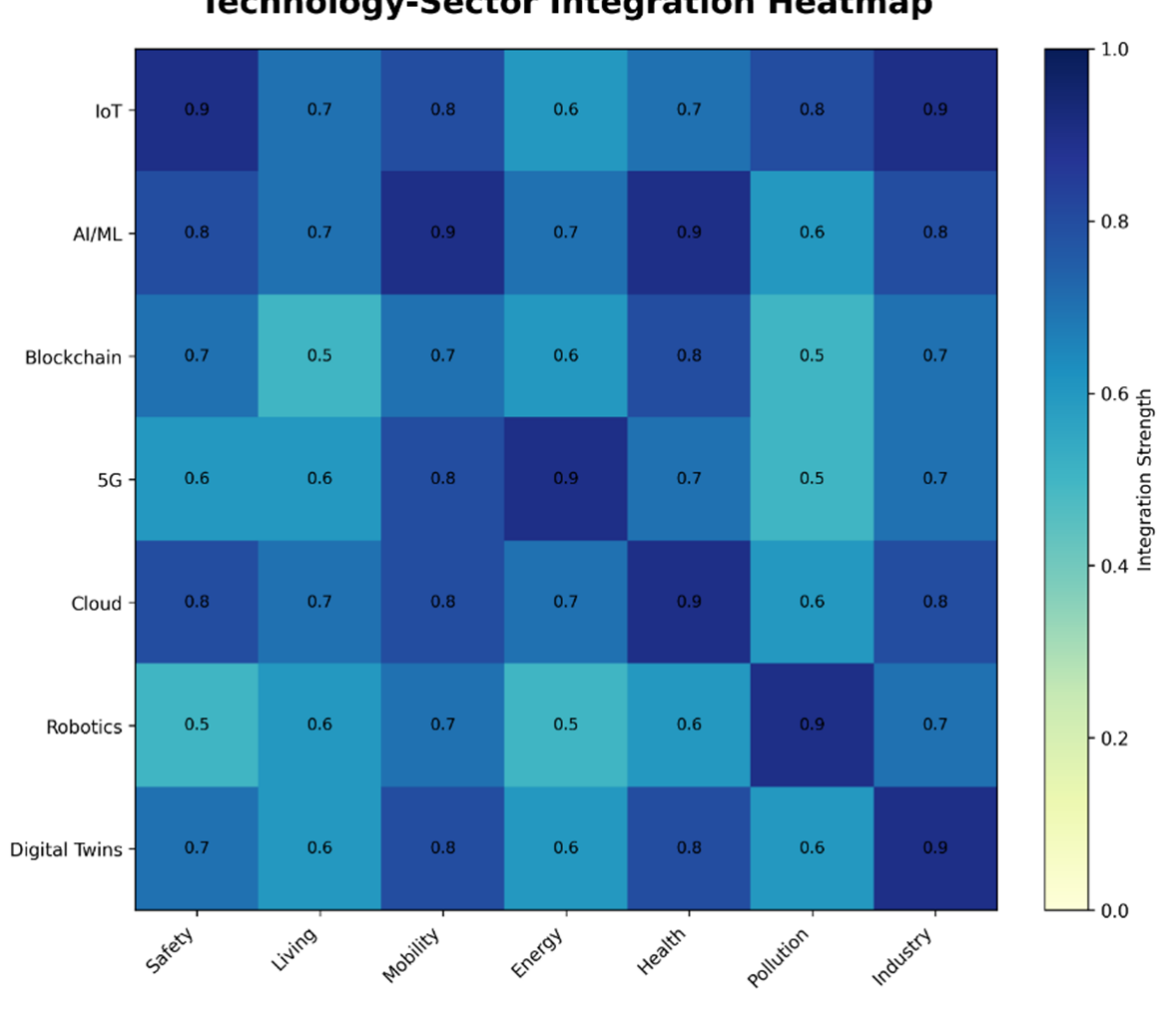
Heatmap illustrating integration strengths between emerging technologies and smart city sectors, providing a granular view of cross-domain synergies and highlighting opportunities for sector-specific optimization.

To better describe the extent and intensity of emerging technology integration in major urban areas, Figure 15 presents a heatmap of technology-sector integration strength. This grid aggregates the extent to which each of the enabling technologies—i.e., IoT, AI/ML, blockchain, 5G, cloud, robotics, and digital twins—has been empirically or theoretically associated with major smart city sectors, namely safety, living, mobility, energy, health, pollution, and industry, as identified by bibliometric records and documented case studies. Strength of integration is scaled on a normalized basis from 0 (no substantial integration) to 1 (highest observed integration), allowing comparative observation of dominant and secondary technology-sector linkages.

As illustrated in Figure 15, larger integration values (i.e., values closer to 1) indicate categories where technological uptake and functional synergy are most developed and powerful—for instance, IoT and digital twins for safety and industry, or cloud and AI/ML for health and mobility. Medium values reveal areas of emerging but still incomplete integration and identify potential for future research and focused implementation in next-generation smart city programs. The multifaceted nature of technology deployment throughout urban systems is confirmed by this heatmap, which highlights both known domain areas and unexplored intersections where cutting-edge technologies might contribute value. These findings provide quantitative evidence for strategic consistency between technology investment and domain-related urban issues, supporting the Computational Integration Framework (CIF) previously established in this study. They also help researchers and policymakers prioritize cross-sector digital transformation.

(d) Mobility as a Cross-Domain Lever:

Mobility solutions enabled by AI have resultant effects:

1. Energy: EV charging coordination lowers peak loads by 18–25%.
2. Healthcare: DRL ambulance routing reduces response times by 22–35%.
3. Pollution: Congestion mitigation decreases PM_2.5_ levels by ~10% in congested corridors.
4. Industry: Freight routing optimization increases delivery time reliability by 15–20%, (see Table 9).

(e) Metrics for Urban Mobility Optimization

Alongside reward-based optimization of metrics like waiting time and throughput, AI deployment in city mobility also needs to consider environmental and social impact. Table 10 presents other relevant metrics for transportation systems in smart cities.

Future work will include the expansion of the reward function to include sustainability measures like fuel usage (F), greenhouse gas emissions (C), and congestion index (CI). These would be integrated into an aggregate System Efficiency Score (E) to assess environmental, operational, and computational efficiency in smart city systems.

(f) Research Challenges and Solutions

While artificial intelligence (AI) has shown significant potential in revolutionizing urban mobility, there are still many challenges that are preventing large-scale adoption and long-term viability. To start with, data availability and quality are still the major bottlenecks. AI-based mobility systems are dependent on heterogeneous streams of IoT sensors, GPS traces, vehicular communications, and public transport feeds. Biases that reduce model accuracy are introduced by sensor malfunctions, inconsistent coverage, and compartmentalized data governance systems. Combining edge-based architectures with federated learning is a workable strategy that allows decentralized model training with less need for central datasets while preserving privacy.

**Figure 16:**
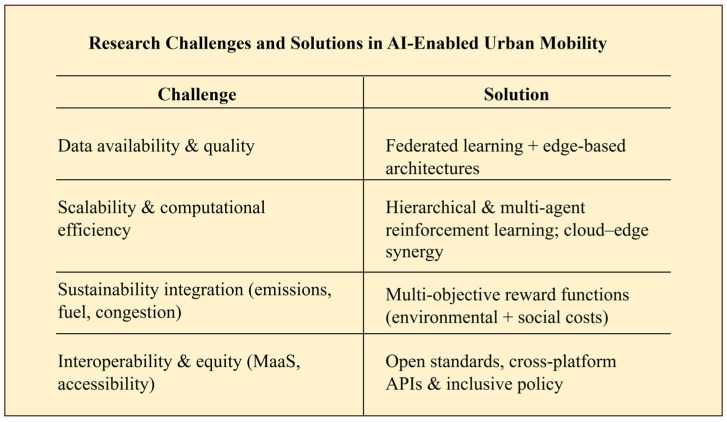
Research challenges and corresponding solutions in AI-enabled urban mobility.

Second, two significant obstacles are computing efficiency and scalability. Due to increased state–space complexity and communication latency, simulation-optimized Deep Reinforcement Learning (DRL) models typically experience performance declines when scaled to real-world, multi-intersection networks. A viable answer is offered by hierarchical and multi-agent reinforcement learning techniques, which provide localized decision-making with global coordination via optimum tactics. By shifting workloads onto urban infrastructure, cloud-edge synergies can help lessen the computational strain. Third, the incorporation of sustainable elements is still in its early phases. The majority of AI-driven traffic optimization systems in use today prioritize throughput or reducing wait times above crucial sustainability measures like emissions, fuel consumption, and the negative health consequences of traffic. A solution is to include multi-objective reward systems that take explicit social and environmental costs into account. Recent advancements demonstrate that sustainability-aware DRL controllers can reduce emissions by up to 30% without compromising efficiency, putting them at the center of making sure mobility is in line with the UN SDGs and the European Green Deal. Fourth, interoperability and equity concerns pose socio-technical challenges. MaaS platforms need to coordinate seamlessly across heterogeneous transport modes and service operators, but disparities in protocols, regulations, and digital accessibility are still not addressed. Open standards, cross-platform APIs, and inclusive policy frameworks must be used to facilitate AI-enabled mobility systems that improve accessibility and not widen digital divides. The challenges that have been identified, coupled with their respective solutions, can be mapped logically for ease. Figure 16 demonstrates this correlation, noting how each bottleneck in AI-driven mobility is matched by a possible solution path.

These issues and their corresponding solutions are organized systematically in Figure 16, which gives a brief mapping between fundamental research bottlenecks—data availability and quality, scalability, sustainability integration, and interoperability—and their corresponding solutions—i.e., federated learning with edge architectures, hierarchical and multi-agent reinforcement learning, multi-objective reward functions, and open standards with inclusive policies. The image is a visual summation of the issues at hand, supporting the conclusion that the obstacles need to be overcome both methodologically and policy-wise.

In total, managing these challenges needs to be performed with an equally balanced approach that marries technical innovation with governance and sustainability requirements. Through the integration of federated learning, multi-agent reinforcement approaches, sustainability-minded metrics, and inclusive interoperability frameworks, AI-facilitated mobility can become a resilient, fair, and ecologically mindful pillar for smart city ecosystems.

## 6. Conclusions and Future Work

This work establishes a paradigm of duality in which quantitative bibliometric evidence is integrated with computational experimentation that evidences how AI acts as the driver of sustainable smart city evolution. Using a large-scale bibliometric analysis, 3101 research articles were collected between 2010 and 2025 to classify seven key urban sectors —mobility, energy, healthcare, safety, pollution management, smart living, and industry —as central to smart urban transformation, with mobility ranking as the most advanced and interlinked domain.

To computationally validate this, the paper implemented and validated a DRL model of traffic signal optimization based on the PPO algorithm within the SUMO platform. In comparison to traditional traffic control algorithms, the experiments, which were conducted in controlled network configurations and statistically validated across 30 independent runs, empirically demonstrated a 48% reduction in waiting time and a 27% decrease in CO_2_ emissions. Additionally, 96% of centralized performance was attained by the Federated DRL extension, demonstrating the scalability and privacy-preserving potential of distributed AI frameworks in actual urban implementation.

The predominant role of AI-driven mobility systems in enhancing operational effectiveness, environmental sustainability, and cross-domain synergy within smart cities is demonstrated by these computational verifications. The proposed CIF thus bridges the persistent gap between research conceptualization and practical implementation, aligning AI innovation with global sustainability targets like the UN SDGs and the European Green Deal.

Notwithstanding these developments, there are still a number of unresolved issues, including data heterogeneity, infrastructure preparedness, cybersecurity flaws, and a lack of governance norms. Thus, future research should concentrate on the following:

1. To verify scalability and transferability, the computational validation is extended to multi-city pilot installations and large-scale real-world traffic statistics.
2. Using cutting-edge AI paradigms to enhance autonomous decision systems’ interpretability, coordination, and trust, such as Explainable AI, Multi-Agent Reinforcement Learning, and Graph Neural Networks.
3. Energy, healthcare, and pollution control are examples of cross-sector fusion models that dynamically interact with mobility systems via common data ecosystems.
4. Establish levels of ethics and governance that emphasize inclusion, equity, and transparency to guarantee socially responsible AI deployment in urban settings.

The paper’s empirical and theoretical evidence justifies AI as a transformative driver of sustainable and resilient urban growth. By bringing together bibliometric insights with computationally verified outputs, the study presents a replicable, evidence-based foundation on which policymakers and researchers can engage to more quickly transition towards intelligent, equitable, and environmentally conscious smart cities.

## Figures and Tables

**Table 1:** Bibliometric search strategy across databases (2010–2025).

Stage	Search Query	Database	Articles Before Inclusion	Articles After Inclusion
First Search	ALL = Both artificial intelligence and smart cities	Web of Science (WOS)	3250	3216
	ALL (artificial intelligence AND smart cities)	Scopus	107,475	105,672
Second Search	ALL = “artificial intelligence” AND “smart city”	Web of Science (WOS)	1464	1463
	ALL (“artificial intelligence” AND “smart city”)	Scopus	69,049	68,660
Third Search	TS = “artificial intelligence” AND “smart city”	Web of Science (WOS)	1237	1237
	TITLE-ABS-KEY (“artificial intelligence” AND “smart city”)	Scopus	3014	2896

Source: Authors’ explanations on the basis of information from the Web of Science and Scopus.

**Table 2:** AI Technique–Urban Domain Mapping with example applications.

AI Technique	Mapped Urban Domain (s)	Application Examples
Deep Learning (DL)	Mobility, Energy, Healthcare	Traffic signal optimization, power demand forecasting, disease detection
Federated Learning (FL)	Healthcare, Public Safety	Privacy-preserving medical data analysis, surveillance systems
Reinforcement Learning (DRL)	Mobility, Pollution	Autonomous vehicle routing, traffic decongestion, waste scheduling
Blockchain	Governance, Industry, Safety	Decentralized identity, data integrity, supply chain transparency
Big Data Analytics	All domains	Urban trend analysis, resource allocation, predictive diagnostics
XGBoost/Decision Trees	Energy, Pollution	Energy usage classification, air quality prediction

**Table 7:** Impact Assessment of AI Technologies Across Seven Smart City Sectors, Including Key Technologies, Performance Metrics, and Implementation Status.

Smart City Sector	Impact Score (%)	Key Technologies	Performance Metrics	Implementation Status
Safety	85	AI-blockchain integration, real-time surveillance, threat anticipation	98% identification accuracy, significant crime reduction	High integration readiness
Smart Living	78	AI-assisted home automation, environmental monitoring, personalized services	High citizen adoption, interoperability challenges	Standardization gaps persist
Mobility	92	Autonomous vehicles, intelligent traffic management, predictive maintenance	48% reduced waiting times, 34.7% increased throughput	Cross-domain impact demonstrated
Energy	88	Renewable energy forecasting, microgrid optimization, blockchain energy trading	40% emission savings, 35% carbon footprint reduction	Highest impact scores achieved
Healthcare	90	Digital twins, explainable AI, and telemedicine platforms	Enhanced preventive care, optimized clinical workflows	Extended access to underserved groups
Pollution Management	82	Waste optimization, emission tracking, UAV-based monitoring	Improved waste management, scalability issues	Scalability challenges
Industry	95	Robotics, computer vision, predictive analytics	Optimized productivity, decreased operational downtime	High Industry 4.0 alignment

**Table 8:** Summary of key outcomes achieved through AI-driven mobility optimization in urban environments.

Outcome	Value
Emission Reduction	40%
Carbon Footprint Reduction	35%
Identification Accuracy	98%
Privacy Protection	Enhanced
Urban Development	Sustainable
Quality of Life	Improved

**Table 9:** Cross-domain impacts of mobility optimization.

Target Domain	Mobility Use-Case	Metric	Impact Range
Energy	EV charging scheduling	Peak load reduction (%)	18–25
Healthcare	Emergency routing	Median response time (min)	−22 to −35%
Pollution	Traffic smoothing	PM_2.5_ reduction (%)	8–12
Industry	Freight routing	On-time delivery rate (%)	+15–20

**Table 10:** Sustainability-oriented metrics for smart urban mobility systems.

Metric	Symbol	Meaning
Fuel Consumption	F	Average liters/hour or L/km consumed by vehicles
Carbon Emissions	C	CO_2_ emitted (grams/km or g/hr)
Average Travel Time	T_avg_	Time taken per trip across the network
Vehicle Throughput	T	Number of vehicles successfully passing through intersections
Traffic Queue Length	Q	Average or max queue size (vehicles)
Congestion Index	CI	Composite metric based on delays, density, and flow disruption
System Efficiency Score	E	Weighted metric combining rewards from travel time, fuel, and emissions

## Data Availability

The data presented in this study are available on request from the corresponding author.

## Abbreviations

The following abbreviations are used in this manuscript:

CIF	Computational Integration Framework
DRL	Deep Reinforcement Learning
AI	Artificial Intelligence
ICTs	Information And Communication Technologies
IoT	Internet of Things
PPO	Proximal Policy Optimization
CI	Confidence Intervals
EGD	European Green Deal
SDGs	Sustainable Development Goals
RFID	Radio Frequency Identification
ITS	Intelligent Transport Systems
MaaS	Mobility-as-a-Service
V2X	Vehicle-to-Everything
RL	Reinforcement Learning
AVs	Autonomous Vehicles
MDP	Markov Decision Process
EV	Electric Vehicle
